# Effect of wheat species (Triticum aestivum vs T. spelta), farming system (organic vs conventional) and flour type (wholegrain vs white) on composition of wheat flour – Results of a retail survey in the UK and Germany – 3. Pesticide residue content

**DOI:** 10.1016/j.fochx.2020.100089

**Published:** 2020-03-29

**Authors:** Juan Wang, Gultakin Hasanalieva, Liza Wood, Christos Anagnostopoulos, Georgios Ampadogiannis, Eleftheria Bempelou, Maroula Kiousi, Emilia Markellou, Per Ole Iversen, Chris Seal, Marcin Baranski, Vanessa Vigar, Carlo Leifert, Leonidas Rempelos

**Affiliations:** a Human Nutrition Research Centre, Institute of Cellular Medicine, Newcastle Upon Tyne NE2 4HH, UK; b School of Agriculture, Food & Rural Development, Nafferton Ecological Farming Group, Newcastle University, Nafferton Farm, Stocksfield, UK; c Department of Sustainable Crop and Food Protection, Faculty of Agriculture, Food and Environmental Sciences, Universita Catollica del Sacro Cuore, I-29122 Piacenza, Italy; d Benaki Phytopathological Institute (BPI), Athens, Greece; e Department of Nutrition, Institute of Basic Medical Sciences, University of Oslo, Oslo, Norway; f Department of Haematology, Oslo University Hospital, Oslo, Norway; g Department of Functional and Organic Food, Institute of Human Nutrition Sciences, Warsaw University of Life Sciences, Nowoursynowska 159c, 027-776 Warsaw, Poland; h Centre for Organics Research, Southern Cross University, Military Rd., Lismore, NSW, Australia

**Keywords:** Pesticides, Organic production, Whole-grain, Common wheat, Spelt wheat

## Abstract

Wheat is a major component of the Northern European diet and contributes significantly to dietary pesticide exposure. Here we report results of a 2-year retail survey, which compared pesticide residues in organic and conventional, whole-grain and white, common and Spelt wheat flour brands available in the UK and Germany. Pesticide residues were detected significantly more frequently in conventional (87%) than organic (25%) flour samples. Chlormequat, a plant growth regulator, was the most frequently detected compound. Total concentrations of pesticide residues were (a) ~4 times higher in conventional than organic, (b) ~100% higher in common than Spelt wheat flour and (c) ~110% higher in conventional whole-grain than white flour samples, but (d) not significantly different in organic whole-grain and white flour. Results suggest that the use of organic wheat products allows increased whole-grain cereal consumption in line with nutritional recommendations, without an increase in dietary pesticide intake.

## Highlights

• Conventional flour had 4-time higher pesticide residues than organic flour.
• Conventional whole-grain flour had 2-time higher pesticide residues than white flour.
• Organic whole-grain and white flours had similarly low pesticide residue levels.
• Common wheat had 2-times higher pesticide residues that Spelt wheat flour.

## 1 Introduction

Wheat (*Triticum aestivum*) is one of the most important cereal species used for human consumption in Europe and North America and the main ingredient in many staple food products such as bread, pasta, noodles, and other bakery products (Goesaert et al., 2005). Currently, the majority of wheat products is made from white flour, where the bran and germ are removed from the endosperm before milling. However, the use of whole-grain flour (where the whole grain is milled), is increasingly recommended by nutritionists, because an increasing number of scientific studies has shown associations between wholegrain consumption and a reduced risk of obesity and a range of chronic diseases including type 2 diabetes, certain cancers and cardiovascular diseases (Cho et al., 2013, Jones and Engleson, 2010). The health benefits from whole-grain consumption are thought to be mainly associated with the higher fibre, mineral and (poly)phenol/antioxidant content (which is mainly in the bran fraction of the grain) of whole-grain flour (Jones and Engleson, 2010, Williamson, 2017). However, there are also studies, which reported the presence of higher concentrations of nutritionally undesirable pesticide residues in bran compared with white flour (Bajwa & Sandhu, 2014).

Organic farming standards prohibit the use of synthetic chemical pesticides, while a wide range of different pesticides (herbicides, fungicides, insecticides and growth regulators) are used in conventional wheat production (Pesticide Action Network UK, 2017, Rempelos et al., 2018). A recent meta-analyses of published comparative data concluded that organic production methods result in lower pesticide residues in cereals/cereal products (Barański et al., 2014). It is therefore important to consider the potential confounding effects of cereal production methods in studies comparing pesticide residues in white and whole-grain cereal products.

Spelt wheat (*Triticum spelta*) was widely used in Northern Europe in the past, but is now considered a minor cereal. Spelt is currently increasing its market share and this is thought to due to (a) its ability to grow under low input conditions (which make it particularly suitable for organic farming systems) and (b) consumer perceptions that spelt wheat has a higher nutritional value than common wheat (Dean et al., 2007). However, due to a lack of scientifically sound comparative studies there is still considerable uncertainty about whether or not and to what extent spelt wheat has a superior nutritional composition (Escarnot, Jacquemin, Agneessens, & Paquot, 2012). Also, there are to our knowledge, no comparative retail surveys in which (a) pesticide residues in common and spelt wheat based cereal products were compared and (b) confounding factors such as sample region (e.g. different countries), wheat farming system (e.g. organic vs conventional) and grain processing method (e.g. white or wholemeal products) were considered in the survey design.

The main objectives of this study were therefore to (a) compare pesticide loads in white and whole-grain common and Spelt wheat flour brands/products available in the UK and Germany (b) quantify the effect of contrasting primary production methods (organic vs conventional) on pesticides loads in wheat flour and (c) identify potential interactions between primary production protocols, wheat species (*T. aestivum* vs *T. spelta*) and post-harvest processing (white vs whole-grain flour) on pesticides loads.

Hypotheses: *The use of organic production methods results in lower pesticide residues in wheat flour than conventional production protocols*. *The effect of using organic production methods on pesticide residue levels is augmented*/*confounded by* (*a*) *crop genetic and* (*b*) *postharvest storage*/*processing*/*milling factors*.

## 2 Methods and materials

### 2.1 Retail survey design

The retail survey of wheat flour for pesticide residue analysis was conducted over two successive years (2016 and 2017) in the UK and in Germany. The experimental design included 4 factors/variables: (1) country (UK and Germany), (2) wheat species (*T. aestivum* vs *T. spelta*), (3) farming system (organic vs conventional) and (4) flour type (white vs wholegrain).

Cereal brands were used as replicates, with only one sample per brand (supermarket own or manufacturers brands) being used for each combination of wheat species, farming system and flour type in each year. This was primarily done to avoid pseudo-replication, since the use of more than one sample per brand could have resulted in both flour samples originating from the same batch of grain used by millers; different brands were assumed to have been made by different mills or at least from different grain batches (see Table S1 for the number of samples/flour brands assessed for different wheat flour types in the 2 countries).

In total, 260 samples were purchased from supermarkets in UK (Tesco, Waitrose, Sainsbury, Marks & Spencer, Holland& Barrett, Fenwick Food Hall, Morrisons) and Germany (Aldi, Budnikowsky, Combi, Demeter, Denn’s Biomarket, Dm, Edeka, Kaufland, LIDI, Nahkauf, Netto, NP-Discount, Reformhaus, Rewe) and websites in UK (Allinson, Amazon, Bacheldre Watermill, Buywholefoodonline, Gilchester online, Matthews Gotswold, Sharpham Park, Shipton Mill online, Wessex Mill) in the same period in each year (see Table S1 for the numbers of brands analysed for each flour type). Flour was unpacked from their original packages and transferred to vacuum food bags then stored in a −80 °C freezer in containers with silica gel until analysis.

### 2.2 Pesticide analysis

Pesticide residue analyses were carried out by the Benaki Phytopathological Institute (Stefanou Delta Street, Kifissia, Athens, 14561, GR) in 2016 and Concept Life Sciences Ltd. (19 Spring Gardens, Manchester, UK; www.conceptlifesciences.com) in 2017 using validated extraction methods and GC-ECD, GC/MS and LCMSMS based analytical protocols. Further information on the analytical protocols is provided in the Supplementary Material. In 2016, flour samples were analysed for 57 different active ingredients. In 2017, flour samples were analysed for 492 active ingredients. Tables S2 to S4 in the supplementary material provide a list of compounds analysed, for each compound and the limit of detection of the analysis method used, the maximum residue level (MRL) set by the EU, the type of product (e.g. insecticide) they are used for and the chemical/CPP group they belong to.

### 2.3 Statistical analysis

Analysis of Variance (ANOVA) derived from linear mixed-effects (lme) models (Pinheiro & Bates, 2000) were used to assess the effects and interactions between factors on measured parameters by using the *‘nlme’* package in R (R Core Team, 2018). Data on (a) the proportion of positive samples (=samples in which crop protection product [CPP] residue above the detection limit of the analytical methods used were found) and (b) the concentrations of crop protection product (CPP) residues were analysed. Samples were collected over two years (2016 and 2017), but white spelt flour was not available in Germany during 2016. Therefore it was not possible to include all experimental factors in a single analysis and two separate 3-factor ANOVAs were carried out: (a) ANOVA 1: wheat species (common vs spelt wheat), farming system (organic vs conventional) and flour types (whole-grain and white) and (b) ANOVA 2: country (UK vs Germany), species (common vs spelt) and farming system (organic vs conventional). The hierarchical nature of the experimental design was designated in the random error structures of the model as: replicate (commercial brand)/year/country/wheat species/farming system (ANOVA 1) and replicate (commercial brand)/yea/country/wheat species/flour type (ANOVA 2). For all parameters it was also checked that the residuals were normally distributed by using the *‘qqnorm’* function in R. Where the distribution of residuals was not normal data were transformed (the specific transformations carried out are described in the tables).

In order to further investigate the significant (*p* < 0.05) interactions between factors, general linear hypothesis tests (Tukey contrasts) were performed using the *‘glht’* function of the *‘multcomp’* package (Bretz, Hothorn, & Westfall, 2011) in R. The experimental design was reflected in the same random error structures used for the lme models. This method is allowing multiple comparisons in unbalanced models with arbitrary error distribution and hence arbitrary data distribution and variance structure. Mann Whitney U non-parametric tests (*‘wilcox.test’* command in R) were used as a supplementary test to assess whether two sample means are equal or not. Means and standard errors of means for the main effect and the interaction effect tables were generated by using the *‘tapply’* function in R. When analysing the mean concentrations of pesticides, the concentration in negative samples (=samples in which no pesticides residue was detected by the analytical methods used) were set at half the concentration of the limit of detection of the analytical method as recommended in previous studies (U.S. Environmental Protection Agency, 2000).

For the estimate the total concentration of CPPs in wheat flour, the sum of deltamethrin, chlormequat, piperonyl butoxide, pirimiphos methyl, and 2-phenylphenol residue was used, since these CPPs were detected in quantifiable concentrations in 4% or more flour samples. Data from all pesticides found in less than 4% of samples were included in the estimate, because use of half the LOD as the concentration for all negative samples would have increased the risk of overestimating total pesticide residues. Although cypermethrin and glyphosate were detected in more than 4% of samples they were not included in the estimate because (a) concentration of cypermethrin residue in some positive samples were below the limit of quantification set by the laboratory and not available and (b) the detection limit for glyphosate was different in the analytical methods used in 2016 and 2017.

## 3 Results

In 2016, eight active compounds used in crop protection products (CPPs) were detected in the 113 flour samples including deltamethrin, chlormequat, piperonyl butoxide, pirimiphos methyl, alpha-cypermethrin, pendimethanil, tebuconazole, and mepiquat. In 2017, seven active compounds used in CPPs were detected in 147 flour samples assessed, including deltamethrin, chlormequat, piperonyl butoxide, pirimiphos methyl, 2-phenylphenol, glyphosate, and chlorpyrifos methyl (see Supplementary Material Tables S2 to S4).

### 3.1 Proportion of flour samples testing positive for pesticide residues

The only CPP residues detected in a substantial proportion of flour samples were chlormequat (detected in 35% of flour samples) and piperonyl butoxide (detected in 20% of flour samples). Other CPP residues detected in a smaller proportion flour samples included glyphosate (detected in 14% of samples), 2-phenylphenol (detected in 8% of samples) and cypermethrin (detected in 13% of samples), and pesticides found in only very few samples included pirimiphos-methyl (detected in 5% of samples), deltamethrin (detected in 4% of samples), mepiquat (detected in 2% of samples), pendimethanlin (detected in 2% of samples,), tebuconazole (detected in 2% of samples), and chlorpyrifos methyl (detected in 0.1% of samples).

Table 1 shows the percentage of samples testing positive for the most frequently detected CPPs, when wheat flour from different countries, wheat species, farming system and flour types were compared. ANOVA was only possible for chlormequat, piperonyl butoxide and the proportion of samples testing positive for at least 1 or multiple pesticide residues (Table 1).

**Table 1:** Main effect means ± SE and p-values for the effects of, and interactions between, country (Germany and UK), wheat species (common vs Spelt wheat), farming system (organic vs conventional) and flour type (white vs whole-grain) on the % of wheat flour samples testing positive for specific crop protection product (CPP), at least 1 CCP and multiple (2 or more) CCP residues.

Factor	chlormequat	Piperonyl butoxide^#^	2-phenyl-phenol^##^	glyphosate^##^	cypermethrin^##^	at least 1 CPP	multiple (>2) CPP residues
*Country*							
Germany (n = 16 )	23 ± 11	27 ± 9	13 ± 13	4 ± 3	15 ± 10	54 ± 11	15 ± 6
UK (n = 15)	45 ± 12	20 ± 6	0	16 ± 12	21 ± 9	57 ± 11	24 ± 9
*Species*							
Spelt wheat (n = 15)	33 ± 12	31 ± 9	0	4 ± 3	25 ± 10	64 ± 10	13 ± 6
Common wheat (n = 16)	35 ± 11	17 ± 5	13 ± 13	16 ± 12	11 ± 8	47 ± 11	24 ± 9
*Farming system*							
Conventional (n = 15)	68 ± 11	36 ± 9	13 ± 13	21 ± 11	9 ± 9	87 ± 6	36 ± 9
Organic (n = 16)	3 ± 2	12 ± 5	0	0	25 ± 9	25 ± 8	4 ± 2
*Flour type*							
White (n = 15)	31 ± 11	20 ± 8	13 ± 13	7 ± 5	7 ± 7	49 ± 11	14 ± 7
Whole-grain (n = 16)	37 ± 12	27 ± 7	0	14 ± 11	26 ± 10	61 ± 10	24 ± 8

ANOVA 1 (p-values)*							
*Main Effects*							
Species (SP)	NS	NS	–	–	–	NS	NS
Farming System (FS)	0.003	0.0438	–	–	–	0.0020	0.0116
Flour type (FT)	NS	NS	–	–	–	NS	NS
*Interactions*	NS	NS	–	–	–	NS	NS

ANOVA 2 (p-values)*							
*Main Effects*							
Country (CT)	NS	NS	–	–	–	NS	NS
Species (SP)	NS	NS	–	–	–	NS	NS
Farming System (FS)	<0.0001	0.0045	–	–	–	<0.0001	0.0028
*nteractions*							
CT × FS	0.03401 ^1^	NS	–	–	–	NS	NS
SP × FS	NS	0.04223 ^2^	–	–	–	NS	NS

^#^, only assessed in 2016; ^##^ assessed in both 2016 and 2017, but data shown are for 2017 only, since there were no positive samples in 2016. ^###^ assessed in both 2016 and 2017, but data shown are for 2016 only, since there were no positive samples in 2016.

^1^see Table 2 for Interaction means ± SE; ^2^ see Table S6 for Interaction means ± SE; * p-values are for ANOVAs carried out on log + 1 transformed data, means and SE presented were calculated using non-transformed data,

Significant main effects were detected only for farming system (Table 1). The percentage of flour samples testing positive for (a) at least 1 CPP, (b) multiple (2 or more) CPP residues, (c) chlormequat, and (d) pirimiphos methyl were significantly higher in conventional compared with organic flour samples (Table 1). Approximately 3-times more conventional than organic flour samples were found to be contaminated with at least 1 CPP and there were 8 times more conventional than organic samples with multiple CPP residues (Table 1). However, cypermethrin was detected in more organic than conventional flour samples (Table 1).

For chlormequat (CCC) a weak, but significant interaction between country and farming system was detected. The difference in the proportion of positive samples between organic and conventional flour was greater in the UK (5% vs 91%) than in Germany (0 vs 47%) (Table 2).

**Table 2:** Interactions means ± SE for the effects of country and farming systems on percentage of samples testing positive for chlormequat.

	Factor 1	Factor 2		
		Farming System		
Crop protection products (CPPs)	Country	Conventional		Organic
Chlormequat	Germany	47 ± 18	A b	0 ± 0 B a
	UK	91 ± 6	A a	5 ± 3 B a

For each parameter assessed means labelled with capital letter within the same row or the same lower case letter within the same column are not significant different (Turkey’s honestly significant difference test P < 0.05);

For piperonyl butoxide a weak, but significant interaction between wheat species and farming system was detected (Table 1). For Spelt wheat, twice the number of positive samples were found in conventional than organic samples (41% vs 22%) but the difference was not significant. For common wheat, ten times more positive samples were found in conventional than organic wheat (30% vs 3%) and the difference was significant (Table S5).

### 3.2 Concentrations of pesticide residues

Sufficient numbers of samples with quantifiable concentrations of individual CPPs to obtain normally distributed data for ANOVA were only available for chlormequat. As a result, factorial ANOVAs to compare the proportion of positive samples and CPP concentrations between wheat species, countries, farming systems and flour types could only be carried out for chlormequat and the estimated mean total concentration of CPP residues (=sum of deltamethrin, chlormequat, piperonyl butoxide, pirimiphos methyl, and 2-phenylphenol residues) (Table 3). Results from non-parametric statistical tests obtained for other individual CPPs are provided in the Supplementary Material Table S6 and Figs. S1–S14.

**Table 3:** Main effect means (±SE) and p-values for the effects, and interaction between, country, cereals species, farming system and flour type on chlormequat and estimated total crop protection product (CPP) residue^#^ concentrations (µg/kg) in wheat flour samples collected in 2016 and 2017.

Factor	Chlormequat*	Estimated total CPP residues* #
*Country*		
Germany (n = 121)	24 ± 4.4	54 ± 6
UK (n = 139)	34 ± 3.6	77 ± 10
*Species*		
Spelt wheat (n = 85)	16 ± 3.0	39 ± 3
Common wheat** (n = 175)	36 ± 3.8	80 ± 9
*Farming system*		
Conventional (n = 125)	55 ± 5.0	110 ± 11
Organic (n = 135)	5 ± 0.3	26 ± 1
*Flour type*		
White (n = 151)	22 ± 2.0	54 ± 4
Whole-grain (n = 109)	40 ± 6.0	84 ± 13
Maximum residue level (MRL) µg/kg (EC 2018)	4000	no MRL
ANOVA 1-results (p-values)*		
*Main Effects*		
Species (SP)	0.0007	0.0001
Farming System (FS)	<0.0001	<0.0001
Flour Type (FT)	0.0114	0.0002
*Interactions*		
SP × FS	NS	0.0037 ^3^
SP × FT	NS	NS
FS × FT	0.0008 ^1^	0.0007 ^1^
SP × FS × FT	NS	NS

ANOVA 2-results (p-values)*		
*Main Effects*		
Country (CT)	<0.0001	<0.0001
Species (SP)	0.0006	0.0003
Farming System (FS)	<0.0001	<0.0001
*Interactions*		
CT × SP	NS	0.0331 ^4^
CT × FS	<0.0001 ^2^	0.0021 ^2^
SP × FS	NS	0.0393 ^3^
CT × SP × FS	NS	NS

*p-values are for ANOVAs carried out on reciprocal (1/x) transformed data; means and SE presented were calculated using non-transformed data.

#sum of all CPP residues (deltamethrin, chlormequat, piperonyl butoxide, pirimiphos methyl, and 2-phenylphenol) that were detected in 4 or more flour samples, except for cypermethrin and glyphosate. Cypermethrin and glyphosate were not included in the sum because (a) the concentration of cypermethrin in some positive samples were not available (concentrations were below the limit of quantification set by the laboratory) and (b) the detection limits for glyphosate was different in the analytical methods used 2016 and 2017. ^1^ see Table 4 for interaction means ± SE; ^2^ see Table 5 for interaction means ± SE; ^3^, see Table 6 for interaction means ± SE; ^4^, see Table S7 for interaction means ± SE

For both chlormequat and total CPP concentrations, significant main effects were detected for all four factors included in the two different 3-factor ANOVAs carried out (Table 3). Significantly higher concentrations of chlormequat and total CPP residues were found in the UK than in Germany, common wheat than spelt wheat, conventional than organic and whole-grain than in white flour samples (Table 3). Chlormequat accounted for just under half of the total detectable CPP residue concentrations and mean chlormequat concentrations were 2 orders of magnitude lower than the MRL (Table 3).

For both chlormequat and total CPP residues, ANOVA detected significant two-way interaction between (a) flour type and farming system, (b) country and farming system (Table 3). Significantly higher concentrations of chlormequat and total CPP residues in whole-grain than white flour were only detected in conventional, but not organic flour samples (Table 4). Also, higher concentrations of both chlormequat and total CPP residues in UK than German flour samples were only detected in conventional, but not organic flour samples (Table 5).

**Table 4:** Interactions means ± SE for the effects of flour type and farming systems on chlormequat and all crop protection products concentration (µg/kg).

	Factor 1	Factor 2	
		Farming System	
Crop protection products (CPPs)	Flour Type	Conventional	Organic
Chlormequat	White	36 ± 4 A b	5 ± 0.4B a
	Whole-grain	88 ± 11 A a	5 ± 0.5B a
Total CPPs	White	79 ± 7 A b	26 ± 0.4B a
	Whole-grain	166 ± 28 A a	27 ± 1B a

For each parameter assessed means labelled with the same capital letter with row and same lower case letter within the column are not significant different (P < 0.05). Pairwise comparisons of means were carried out on reciprocal transformed data, means and SE presented were calculated with non-reciprocal transformed data.

**Table 5:** Interactions means ± SE for the effects of country and farming systems on chlormequat and all crop protection products concentration (µg/kg).

	Factor 1	Factor 2	
		Farming System	
Crop protection products (CPPs)	Country	Conventional	Organic
Chlormequat	Germany	46 ± 9 A b	5 ± 0B a
	UK	62 ± 6 A a	6 ± 1B a
Total CPPs	Germany	88 ± 10 A b	25 ± 0B a
	UK	128 ± 19 A a	27 ± 1B a

For each parameter assessed means labelled with the same capital letter with row and same lower case letter within the column are not significant different (P < 0.05). Pairwise comparisons of means were carried out on reciprocal transformed data, means and SE presented were calculated with non-reciprocal transformed data.

For total CPP residues, ANOVA also detected significant two-way interaction between (a) wheat species and farming system (Table 3). Significantly higher concentrations of total CPP residues in common than Spelt wheat were only detected in conventional, but not organic flour samples (Table 6).

**Table 6:** Interactions means ± SE for the effects of wheat species and farming systems on all crop protection products concentration (µg/kg).

	Factor 1	Factor 2	
		Farming System	
Crop protection products (CPPs)	Species	Conventional	Organic
Total CPPs	Spelt wheat	60 ± 7 A b	27 ± 1.0B a
	Common wheat	128 ± 15 A a	26 ± 0.4B a

For each parameter assessed means labelled with the same capital letter with row and same lower case letter within the column are not significant different (P < 0.05). Pairwise comparisons of means were carried out on reciprocal transformed data; means and SE presented were calculated with non-reciprocal transformed data.

## 4 Discussion

### 4.1 Limitations of retail survey

Retail surveys such as the one reported here can provide an accurate estimate of (a) the mean nutritional composition of different types of flour available to consumers and (b) the effect of using certified organic production methods on pesticide residue levels in flour. However, information on location of farms, crop varieties and specific agronomic practices used by organic and conventional farmers and post-harvest drying, storage, cleaning, and milling protocols used for organic and conventional grains were not recorded and it was therefore not possible to assess to what extent these parameters affected the flour composition parameters measured. In the discussion we have therefore related the results of the survey reported here to previous experimental and farm survey based studies that focused on identifying climatic, crop genetic, agronomic, grain processing and quality assurance related parameters affecting pesticide residue levels in crops.

### 4.2 Potential risks associated with pesticide residues

The CPP residue present at detectable levels in wheat flour were (a) the plant growth regulators chlormequat and mepiquat (both quaternary ammonium compounds), (b) glyphosate (an organophosphorus herbicide and crop desiccant), (c) pendimethalin (a dinitroaniline herbicide), (d) chlorpyrifos-methyl and pirimiphos-methyl (both organophosphorus insecticides), (e) deltamethrin and alpha-cypermethrin (both synthetic pyrethroid insecticides), (f) piperonyl butoxide (a pesticide synergist which is included in many pyrethrum or synthetic pyrethroid insecticide-based CPPs; it enhances the activity of insecticides by inhibiting insect enzymes which break down insecticides), (g) tebuconazole (a triazole fungicide) and (h) 2-phenylphenol (a fungicide usually used post-harvest (e.g. for disinfection of surfaces and seed boxes) (NPIC, 2019). These compounds are all licensed for use in conventional farming systems in the EU and the concentrations of residues of CPPs detected in wheat flour samples were all below the maximum residue levels (MRLs) set by the EU for the respective individual active ingredients detected (CPPs) (European Commission, 2018).

Although residues below the maximum residue level (MRL) set for specific active ingredients in crop protection products (CPPs) are considered safe by regulators, concern have recently been raised about negative public health impacts of chronic dietary exposure to below MRL-levels of CPPs (Blair, Ritz, Wesseling, & Freeman, 2015). These concerns are based on the realisation that (a) consumers are exposed to mixtures of CPPs in food, while regulatory safety evaluations only require toxicity testing of individual active compounds (Hernández et al., 2013, Kjeldsen et al., 2013) and (b) many of the most widely used CPPs were shown to be endocrine disrupting chemicals (EDCs) (Mnif et al., 2011).

Nearly all CPPs detected in wheat flour in this study (chlormequat, chlorpyrifos-methyl, glyphosate, phenyl-phenol, piperonyl butoxide, cypermethrin, deltamethrin, tebuconazole) are suspected or confirmed EDCs (European Food Safety Authority, 2015, Mnif et al., 2011).

Like hormones, EDCs can have physiological impacts at very low concentrations and there is evidence from both *in vitro* and animal studies demonstrating additive effects of exposure to mixtures of EDCs (Petrakis et al., 2017, Yang et al., 2015). EDCs can also have U-shaped or inverted U-shaped, non-monotonic dose-response curves (Petrakis et al., 2017, Yang et al., 2015) and exposure can result in epigenetic alterations and programming which may eventually lead to an increased incidence of a variety of diseases (including obesity, type-2 diabetes, immune abnormalities and cancer) later in life or in subsequent generations (Petrakis et al., 2017, Yang et al., 2015). This makes it virtually impossible to detect their physiological impacts in the one generation animal (usually rodent) model-based toxicity tests required as part of the regulatory pesticide approval process (Petrakis et al., 2017, Yang et al., 2015). The testing of effects of exposure to complex mixtures of pesticides is not usually required as part of the EU pesticide approval process and epigenetic effects of ECDs can only be accurately identified by extensive, multigenerational animal studies, which are also not required as part of standard regulatory pesticide safety testing protocols (Matthiessen et al., 2017).

### 4.3 Effect and potential impacts of organic production methods

Data from this study suggest that switching to organic cereal production methods will result in substantial reduction in chronic dietary exposure to chlormequat (CCC), and total and multiple CPP residues. Considering that (a) cereal products account for between a quarter (e.g. in Germany and the UK) and half (e.g. in China and India) of daily calorie intake (National Geographic, 2019) and (b) most of the CPPs detected in wheat were suspected or confirmed EDCs, switching to organic cereal products could potentially have significant positive health impacts.

Recent epidemiological studies from the US suggest that this may particularly apply to reproductive health, since reduced dietary intake of CPPs via fruit and vegetables was reported to be linked to higher sperm quality in men (Chiu et al., 2015) and higher pregnancy rates among women undergoing infertility treatment (Chiu et al., 2018). Dietary exposure to EDCs has also been linked to attention deficit disorder (ADHD), Parkinson’s and Alzheimer’s disease, obesity, diabetes, hypospadias and cancer (Burns et al., 2013, Petrakis et al., 2017, Yang et al., 2015). The 4-fold reduction in dietary CPPs intake and lower exposure to multiple CPPs with organic cereals may also partially explain the results of recent cohort studies comparing health outcomes in individuals with low and high levels of organic food consumption. These studies reported significant positive associations between high levels of organic food consumption and lower risks of obesity, metabolic syndrome, pre-eclampsia and eczema, hypospadias and cancer (Baranski et al., 2017, Baudry et al., 2018). In addition, CCC residues, at concentrations found in conventional wheat in the UK, were recently shown, to be a major driver for differences in plasma hormone profiles and lymphocyte proliferation (a marker for immune system responsiveness) detected between rats raised on organic and conventional feeds (Średnicka-Tober et al., 2013).

CCC is used in cereals production to reduce stem length/longitudinal shoot growth and thereby increasing lodging resistance and improving harvest index and yield (EFSA, 2008). The MRL set by the EU for chlormequat is 4 mg/kg which ~100 time higher than the concentrations found in flour samples in this study. However, dietary exposure to CCC was described to increase the risk of liver damage, tumours, and reduced reproductive and foetal health and fertility in animal models, pigs and/or humans (Huang et al., 2016, Li et al., 2011, Sørensen and Danielsen, 2006). The Danish pig industry recommended limiting the use of grain from crops treated with chlormequat and other growth regulators for breeding stock in 1990 and evidence that CCC negatively affects fertility in experimental animals at concentrations that are below the acceptable intake emerged more than 10 years ago (Sørensen & Danielsen, 2006). However, CCC is still permitted for use in cereal crops for human consumption in most EU-countries.

The application of chlormequat, piperonyl butoxide and cypermethrin (which were detected as residues in organic flour samples) to cereal crops is prohibited under organic farming standards (Pesticide Action Network UK, 2017). However, in some European countries, derogations can be obtained from organic certification bodies for the use of cypermethrin and piperonyl butoxide based CPPs for insect control in grain storage facilities that are subsequently (following a defined time period after CPP application) used for storing organic cereal grain. The finding of these compounds in organic crops could therefore be due to (a) cross contamination from neighbouring conventional fields, (b) permitted use of pyrethroid based insecticide products in grain stores and/or (c) illegal use of synthetic chemical CPPs by organic producers.

The finding that a higher proportion of organic (25%) than conventional (5%) flour samples tested positive for residues of alpha-cypermethrin residues should be further investigated. This together with the finding of piperonyl-butoxide (an activity enhancer often uses in pyrethroid formulations) in 12% of organic flour samples may indicate the use of synthetic pyrethroid products in the field or in grain storage facilities. Different to pyrethrum-CPPs (which are based on plant extract-based “natural” pyrethrins as active ingredients), synthetic pyrethoid-based CPPs are not approved for use in organic farming (Pesticide Action Network UK, 2017).

### 4.4 Effect of wheat species

However, the finding that Spelt wheat flour had only half the CCC and total CPP residue concentration compared with common wheat flour, could have been due to either (a) fewer CPPs having been applied to Spelt crops and/or (b) the husk surrounding the Spelt grain having provided partial protection against the grain becoming contaminated. Spelt wheat is often described as a more stress resistant, robust species than modern common wheat cultivars (Rüegger & Winzeler, 1993). Also, protection by the husks is known to contribute to the higher levels of resistance in Spelt wheat against mycotoxins (Vučković, Bodroža-Solarov, Vujić, Bočarov-Stančić, & Bagi, 2013).

A significant difference in CPP residues between Spelt and common wheat was only detected when conventional flour samples were compared, which suggests that switching from common to Spelt wheat consumption would result in a 50% reduction the total dietary CPP residue intake only for conventional but not organic food consumers.

### 4.5 Effect of flour type

There is increasing evidence for significant health benefits of switching from white to whole-grain cereal consumption (Jones & Engleson, 2010). However, conventional whole-grain flour contained more than 2-times higher CCC and CPP-residues than conventional white flour, which is consistent with previous studies, that reported higher concentrations of pesticides in whole-grain compared with white flour food products (Bajwa & Sandhu, 2014).

In contrast, CCC and total CPP residues concentrations in organic flour were not only substantially lower, but also similar in whole-grain and white flour. This clearly demonstrates that organic whole-grain cereal consumption allows the nutritional benefits of whole-grain consumption (higher fibre, mineral and (poly)phenol/antioxidant intakes) to be achieved without a simultaneous increase in dietary exposure to pesticide residue.

### 4.6 Effect of country

The slightly higher CPP residues in conventional UK compared with German wheat may suggest that the CPP spraying regimes used in Germany are less intensive than those used in the UK. However, in retail surveys such as the one reported here it is not possible to obtain information on the agronomic protocols and especially crop protection practices used to produce the wheat grain from which the flour was made. Also, a proportion of the grain used to produce flour is imported in both Germany and the UK, but for most brands it was not possible to identify whether or not they used imported grain, from the information provided on the label. As a result, it was not possible to identify the reasons for the differences in CPP-residue levels between the two countries.

## 5 Conclusion

Results from this study support the hypothesis that organic cereal production methods result in significantly lower pesticide residues in wheat flour and suggest that switching to organic wheat flour product may substantially reduce dietary exposure to total CPP residues. However, since residue levels in all retail flour products were below the MRLs set for specific active ingredients/substances used in CPPs they are currently considered safe by EU and national regulators.

Results also provide evidence for the hypothesis that crop genetic and postharvest processing parameters affect and/or augments the impact of primary production protocols (organic vs conventional) on pesticide residue levels. Most importantly, whole-grain flour was shown to have substantially higher pesticide residues than white flour, but only when conventional flour samples were compared.

Due to the uncertainty about the potential effects of exposure to mixtures of CPP residues and the lack of testing for epigenetic effects of exposure to endocrine disrupting CPP there is an urgent need to investigate the potential physiological and health impact of reducing pesticide exposure via switching to organic food consumption. There is a particular need for controlled dietary intervention studies with animal models and humans, to provide a mechanistic understanding for associations between organic food consumption and positive health outcomes associated with organic food consumption reported in recent human cohort studies (Baranski et al., 2017, Baudry et al., 2018).

## CRediT authorship contribution statement

**Juan Wang:** Conceptualization, Data curation, Formal analysis, Investigation, Methodology, Visualization, Writing - original draft, Writing - review & editing. **Gultakin Hasanalieva:** Investigation, Methodology. **Liza Wood:** Investigation. **Christos Anagnostopoulos:** Investigation, Methodology, Writing - review & editing. **Georgios Ampadogiannis:** Methodology, Writing - review & editing. **Eleftheria Bempelou:** Investigation. **Maroula Kiousi:** Investigation. **Emilia Markellou:** Conceptualization, Writing - review & editing. **Per Ole Iversen:** Writing - review & editing. **Chris Seal:** Methodology, Project administration, Resources, Supervision, Validation, Writing - review & editing. **Marcin Baranski:** Data curation, Formal analysis, Validation. **Vanessa Vigar:** Writing - review & editing. **Carlo Leifert:** Conceptualization, Funding acquisition, Project administration, Resources, Supervision, Validation, Writing - original draft, Writing - review & editing. **Leonidas Rempelos:** Data curation, Formal analysis, Investigation, Supervision, Visualization, Writing - original draft, Writing - review & editing.

## Declaration of Competing Interest

The authors declare that they have no known competing financial interests or personal relationships that could have appeared to influence the work reported in this paper.
