# Supplementary material for: Effect of wheat species (Triticum aestivum vs T. spelta), farming system (organic vs conventional) and flour type (wholegrain vs white) on composition of wheat flour – Results of a retail survey in the UK and Germany – 3. Pesticide residue content
Source: Food Chem X. 2020 Mar 29;7:100089. doi: 10.1016/j.fochx.2020.100089 (PMC7327242; doi:10.1016/j.fochx.2020.100089)
Supplement: Supplementary data 1 [file mmc1.docx]

**Supplementary material**

**Material and methods**

***Pesticide extraction and analysis***

Due to the contrasting physicochemical properties of the pesticides, 4 different extraction methods were used for (1) multi-residues; (2) 2.4D and fluazifop; (3) chlormequat and mepiquat; and (4) glyphosate, respectively, and 3 different analyses methods (GC-ECD, GC/MS and LCMSMS) were applied. Most pesticides were analysed a multi-residue method (see section 2.2.1 below), except in the cases of 2.4D, fluazifop, chlormequat, and mepiquat (see below).

1 Multi-residue method: For the extraction of the majority of the analyses, the protocol of QuEChERS method concerning commodities with high fat content was followed as described previously (C. J. Anagnostopoulos, Aplada Sarli, Miliadis, & Haroutounian, 2010; C. J. Anagnostopoulos & Miliadis, 2009). The LC-MS/MS analysis was performed by an Agilent Series 1200 liquid chromatograph equipped with a reverse phase Zorbax Eclipse XDB C18 3.5μm particle size, 150mm x 2.1mm analytical column (Varian, Palo Alto, CA, USA). Detection was achieved using a triple quadrupole mass spectrometer (Agilent Triple Quad 6410) equipped with an electrospray ionization interface operating in positive mode. For the GC analysis, pesticides were separated and determined in two Agilent 6890 gas chromatographs, with a splitless injectors equipped with a DB-5-MS column (30 m, 0.32mm i.d. and 0.25 μm film thickness) and a DB-17 MS column (30 m, 0.3mm i.d. and 0.25 μm film thickness) each connected to an ECD detector.

2. Analysis of 2.4D and fluazifop: For the extraction of *2.4D* and *fluazifop* a different variation of the QuEChERS method for acidic pesticides was adopted (European Commission, 2007). In this variation, before addition of acetonitrile, 300μL of 5N NaOH solution were added to adjust the pH to 12. The tube was shaken vigorously for 1min and the mixture was left to stand for 30min. Then 300μL of 5N H_2_SO_4_ solution and acetonitrile was added. Detection was performed with LCMSMS (Chris J. Anagnostopoulos, Liapis, Haroutounian, & Paspatis, 2013){Anagnostopoulos, #122}.

3. Analysis of chlormequat, mepiquat: For the extraction of *chlormequat* and *mepiquat,* the QuPPe protocol was used (Anastassiades et al., 2016) and the determination was conducted using LCMSMS (Danezis, Anagnostopoulos, Liapis, & Koupparis, 2016).

4 Analysis of glyphosate: Similar for the extraction of glyphosate, the QuPPe protocol was used (Anastassiades et al., 2016) in combination with FMOC derivatization as follows: An aliquot of 5 ± 0.05 g was weighted in a 50 mL centrifuge tube and an appropriate amount of HPLC water was added in a ratio ranging from 1:2 to 1:3, depending on the product. The homogenization time depended on the size and nature of the sample. The sample slurry was extracted with 10 mL of methanol. The mixture was shaken using an orbital shaker for 2 hours (in case of a big batch of samples) or using an Ultra Turrax (T25 Basic Ultra Turrax) for 3 min. (in case of individual samples) and centrifuged at 4000 rpm for 5 min. An aliquot of 5 mL was transferred into a 15 mL plastic centrifuge tube and let in the freezer for at least 2 hours or overnight. For the derivatization, an aliquot of 2 mL was transferred into a 15 mL plastic centrifuge tube and 1 mL borate buffer (pH=9) and 0.5 mL of 6000 ppm FMOC solution was allowed to react for 10min. at 70^o^C. Before injecting in the chromatographic system, the final solution was filtered through a 0.22 μm disposable Cellulose syringe filter. The **LC-MS/MS** analysis was performed by an Agilent Series 1200 liquid chromatograph equipped with a reverse phase Zorbax Eclipse XDB C_18_ 3.5μm particle size, 150mm x 2.1mm analytical column (Varian, Palo Alto, CA, USA). Detection was achieved using a triple quadrupole mass spectrometer (Agilent Triple Quad 6410) equipped with an electrospray ionization interface operating in negative mode. The identification of glyphosate was based on retention time (R.T.= 15.4) and the presence of coinciding peaks for two selective transitions (m/z 390 → 168 and 390 → 160) in the correct abundance ratio.

| ***Table S1.*** Number (n) of wheat flour brands sampled and analysed for pesticide in retail surveys in the UK and Germany in 2016 and 2017. | | | | | | |
| --- | --- | --- | --- | --- | --- | --- |
| **Wheat species** | **Farming** | **Flour** |  | **No. of flour samples** | | |
| **Country** | **system** | **type** |  | **2016** | **2017** | **TOTAL** |
| **Common** |  |  |  |  |  |  |
| **wheat flour** | | |  |  |  |  |
| **Germany** | **Conventional** | **White** |  | 12 | 12 | **24** |
|  |  | **wholemeal** |  | 3 | 6 | **9** |
|  | **Organic** | **White** |  | 9 | 12 | **21** |
|  |  | **wholemeal** |  | 6 | 8 | **14** |
| **UK** | **Conventional** | **White** |  | 15 | 24 | **39** |
|  |  | **wholemeal** |  | 11 | 10 | **21** |
|  | **Organic** | **White** |  | 11 | 16 | **27** |
|  |  | **wholemeal** |  | 10 | 10 | **20** |
|  | **TOTAL common wheat** | |  | **77** | **98** | **175** |
|  |  |  |  |  |  |  |
| **Spelt wheat flour** | | |  |  |  |  |
| **Germany** | **Conventional** | **White** |  | 7 | 8 | **15** |
|  |  | **wholemeal** |  | 3 | 5 | **8** |
|  | **Organic** | **White** |  | 4 | 11 | **15** |
|  |  | **wholemeal** |  | 6 | 9 | **15** |
| **UK** | **Conventional** | **White** |  | 0 | 2 | **2** |
|  |  | **Wholemeal** |  | 5 | 2 | **7** |
|  | **Organic** | **White** |  | 4 | 4 | **8** |
|  |  | **Wholemeal** |  | 7 | 8 | **15** |
|  | **TOTAL Spelt wheat** | |  | **36** | **49** | **85** |
|  |  |  |  |  |  |  |
| **TOTAL NUMBER OF FLOUR SAMPLES:** | | |  | **113** | **147** | **260** |

| ***Table S2.*** List of pesticide compounds analysed in both 2016 and 2017 and their EU-maximum residue limit (MRL), limit of detection (LOD) in the analytical tests, product type, chemical group and approval status in the EU. | | | | | | | | |
| --- | --- | --- | --- | --- | --- | --- | --- | --- |
|  | **EU MRL** | **LOD* (mg/kg)** | | **No. of positive samples** | | **Type of product ^b^** | **Chemical Group ^b^** | **Approval status in the EU ^a^** |
| **Compound** | **wheat ^a^** | **2016** | **2017** | **2016** | **2017** |  |  |  |
| **2-phenylphenol** | 0.02 | 0.01 | 0.01 | - | **12** | Fungicide | Phenol | A ^1, 2^ |
| Acetamiprid | 0.10 | 0.01 | 0.01 | - | - | Insecticide | Chloropyridinyl neonicotinoids | A |
| Azoxystrobin | 0.50 | 0.01 | 0.01 | - | - | Fungicide | Strobilurin | A |
| Bifenthrin | 0.50 | 0.01 | 0.01 | - | - | Insecticide, Acaricide | Pyrethroid | A ^3^ |
| Boscalid | 0.80 | 0.10** | 0.01 | - | - | Fungicide | Carboxamide | A |
| Carbendazim | 0.10 | 0.01 | 0.01 | - | - | Fungicide | Benzimidazole | NA ^1^ |
| **Chlormequat** | **7.00** | 0.01 | 0.01 | **25** | **66** | Plant growth regulator | Quarternary ammonium compound | A |
| Chlorpropham | 0.01 | 0.02** | 0.01 | - | - | Herbicide, Plant growth regulator | Carbamate | A |
| Chlorpyrifos | 0.50 | 0.01 | 0.01 | - | - | Insecticide, Acaricide | Organophosphate | A |
| **Chlorpyrifos-methyl** | **0.05** | 0.01 | 0.01 | - | **1** | Insecticide, Acaricide | Organophosphate | A |
| Clothianidin | 0.02 | 0.01 | 0.01 | - | - | Insecticide | Neonicotinoid | NA |
| Cyfluthrin | 0.04 | 0.01 | 0.01 | - | - | Insecticide, Acaricide | Pyrethroid | NA |
| **Cypermethrin** | **2.00** | 0.01 | 0.01 | **15** | **-** | Insecticide, Acaricide | Pyrethroid | A |
| Cyproconazole | 0.10 | 0.01 | 0.01 | - | - | Fungicide | Triazole | A |
| Cyprodinil | 0.50 | 0.01 | 0.01 | - | - | Fungicide | Anilinopyrimidine | A |
| **Deltamethrin** | **1.00** | 0.01 | 0.01 | **5** | **5** | Insecticide | Pyrethroid | A |
| Diazinon | 0.01 | 0.01 | 0.01 | - | - | Insecticide, Acaricide | Organophosphate | NA |
| Difenoconazole | 0.10 | 0.01 | 0.01 | - | - | Fungicide | Triazole | A |
| * LOD, limit of detection. **, the LOD was assumed to be the same as the limit of quantification (LOQ; the lowest concentration at which the analyte can not only be reliably detected but also reliably quantified) defined by laboratory carrying out the analysis. **^a^**, European Commission (2018) <https://ec.europa.eu/food/plant/pesticides/eu-pesticides-database/public/?event=activesubstance.selection&language=EN>; A, Approved for use in the EU; NA, not approved for use in the EU; ND, MRL not defined by the EU because it is not a plant protection product or not included in the pesticide database; **^b^**, PPDB (2019) <https://sitem.herts.ac.uk/aeru/ppdb/en/atoz.htm>  **^1^** Not permitted for use as a food additive but allowed as a post-harvest treatment (in 4 EU countries only). **^2^** Only uses as insecticide in greenhouses with a permanent structure may be authorised. | | | | | | | | |

| ***Table S2 cont.*** List of pesticide compounds analysed in both 2016 and 2017 and their EU-maximum residue limit (MRL), limit of detection (LOD) in the analytical tests, product type, chemical group and approval status in the EU. | | | | | | | | |
| --- | --- | --- | --- | --- | --- | --- | --- | --- |
|  | **EU MRL** | **LOD* (mg/kg)** | | **No. of positive samples** | | **Type of product ^b^** | **Chemical Group ^b^** | **Approval status in the EU ^a^** |
| **Compound** | **wheat ^a^** | **2016** | **2017** | **2016** | **2017** |  |  |  |
| Dimethoate | 0.05 | 0.01 | 0.01 | - | - | Insecticide, Acaricide | Organophosphate | A |
| Fenhexamid | **0.01** | 0.01 | 0.01 | - | - | Fungicide | Fenhexamid | A |
| Fenpropimorph | 0.15 | 0.01 | 0.01 | - | - | Fungicide | Morpholine | A |
| Fenvalerate | 0.20 | 0.01 | 0.01 | - | - | Insecticide, Acaricide | Pyrazolium | NA |
| Folpet | 0.40 | 0.01 | 0.01 | - | - | Fungicide | Phthalimide | A |
| **Glyphosate** | **10.0** | **3.00**** | **0.01** | - | **21** | Herbicide | Phosphonoglycine | A |
| Imazalil | 0.05 | 0.01 | 0.01 | - | - | Fungicide | Imidazole | A |
| Imidacloprid | 0.10 | 0.01 | 0.01 | - | - | Insecticide | Neonicotinoid | A |
| Metalaxyl | 0.01 | 0.01 | 0.01 | - | - | Fungicide | Phenylamide | A |
| Metribuzin | 0.10 | **0.02**** | **0.01** | - | - | Herbicide | Triazinone | A |
| Omethoate | 0.01 | 0.01 | 0.01 | - | - | Insecticide, Acaricide | Organophosphate | NA |
| Pendimethalin | 0.05 | 0.01 | 0.01 | **3** | - | Herbicide | Dinitroaniline | A |
| **Piperonyl butoxide** | **ND** | 0.01 | 0.01 | **31** | **22** | Synergist **^1^** | Cyclic aromatic | ND |
| Pirimicarb | 0.05 | 0.01 | 0.01 | - | - | Insecticide | Carbamate | A |
| Pirimicarb-desmethyl | **ND** | 0.01 | 0.01 | - | - |  |  | ND |
| Pirimiphos-ethyl | 0.01 **^2^** | 0.01 | 0.01 | - | - | Insecticide | Organophosphate | NA |
| **Pirimiphos-methyl** | **5.00** | 0.01 | 0.01 | **3** | **10** | Insecticide | Organophosphate | A |
| Propiconazole | 0.09 | 0.01 | 0.01 | - | - | Fungicide | Triazole | NA |
| * LOD, limit of detection. **, the LOD was assumed to be the same as the limit of quantification (LOQ; the lowest concentration at which the analyte can not only be reliably detected but also reliably quantified) defined by laboratory carrying out the analysis. **^a^**, European Commission (2018) <https://ec.europa.eu/food/plant/pesticides/eu-pesticides-database/public/?event=activesubstance.selection&language=EN>; A, Approved for use in the EU; NA, not approved for use in the EU; ND, MRL not defined by the EU because it is not a plant protection product or not included in the pesticide database; **^b^**, PPDB (2019) <https://sitem.herts.ac.uk/aeru/ppdb/en/atoz.htm>  **^1^** synergist compound which has no pesticidal activity, but enhances the activity of certain pesticides such as carbamates and pyrethroids;  **^2^** Default MRL of 0.01 mg/kg according to Art 18(1)(b) Reg 396 / 2005. | | | | | | | | |

| ***Table S2 cont.*** List of pesticide compounds analysed in both 2016 and 2017 and their EU-maximum residue limit (MRL), limit of detection (LOD) in the analytical tests, product type, chemical group and approval status in the EU. | | | | | | | | |
| --- | --- | --- | --- | --- | --- | --- | --- | --- |
|  | **EU MRL** | **LOD* (mg/kg)** | | **No. of positive samples** | | **Type of product ^b^** | **Chemical Group ^b^** | **Approval status in the EU ^a^** |
| **Compound** | **wheat ^a^** | **2016** | **2017** | **2016** | **2017** |  |  |  |
| Pyraclostrobin | 0.20 | 0.01 | 0.01 | - | - | Fungicide, Plant growth regulator | Strobilurin | A |
| Pyrethrins | 3.00 | 0.01 | 0.01 | - | - | Insecticide | Plant derived | A |
| Pyrimethanil | 0.05 | 0.01 | 0.01 | - | - | Fungicide | Anilinopyrimidine | A |
| Spiroxamine | 0.05 | 0.01 | 0.01 | - | - | Fungicide | Morpholine | A |
| Tau-Fluvalinate | 0.05 | 0.01 | 0.01 | - | - | Insecticide | Synthetic pyrethroid | A |
| Tebuconazole | 0.30 | 0.01 | 0.01 | **2** | - | Fungicide | Triazole | A |
| Tefluthrin | 0.05 | **0.02**** | **0.01** | - | - | Insecticide | Pyrethroid | A |
| Thiacloprid | 0.10 | 0.01 | 0.01 | - | - | Insecticide | Neonicotinoid | A |
| Thiamethoxam | 0.05 | 0.01 | 0.01 | - | - | Insecticide | Neonicotinoid | A |
| Thiophanate-methyl | 0.05 | 0.01 | 0.01 | - | - | Fungicide | Benzimidazole | A |
| Triadimenol | 0.10 | 0.01 | 0.01 | - | - | Fungicide | Triazole | A |
| * LOD, limit of detection. **, the LOD was assumed to be the same as the limit of quantification (LOQ; the lowest concentration at which the analyte can not only be reliably detected but also reliably quantified) defined by laboratory carrying out the analysis. **^a^**, European Commission (2018) <https://ec.europa.eu/food/plant/pesticides/eu-pesticides-database/public/?event=activesubstance.selection&language=EN>; A, Approved for use in the EU; NA, not approved for use in the EU; ND, MRL not defined by the EU because it is not a plant protection product or not included in the pesticide database; **^b^**, PPDB (2019) <https://sitem.herts.ac.uk/aeru/ppdb/en/atoz.htm> | | | | | | | | |

| ***Table S3.*** List of pesticide compounds analysed in 2016 and their EU-maximum residue limit (MRL), limit of detection (LOD) in the analytical tests, product type, chemical group and approval status in the EU | | | | | | |
| --- | --- | --- | --- | --- | --- | --- |
|  | **EU MRL for** | **LOD*** | **No. of** |  |  | **Approval status** |
| **Compound** | **Wheat ^a^** | **(mg/kg)** | **positive samples** | **Type of product ^b^** | **Chemical Group ^b^** | **in the EU ^a^** |
| 2,4-D | 2.00 | 0.01 | - | Herbicide, Plant growth regulator | Alkylchlorophenoxy | A |
| **2-phenylphenol** | **0.02** | 0.01 | - | Fungicide | Phenol | A^1^ |
| Acetamiprid | 0.10 | 0.01 | - | Insecticide | chloropyridinyl neonicotinoids | A |
| Azoxystrobin | 0.50 | 0.01 | - | Fungicide | Strobilurin | A |
| Bifenthrin | 0.50 | 0.01 | - | Insecticide, Acaricide | Pyrethroid | A^2^ |
| Boscalid | 0.80 | 0.10** | - | Fungicide | Carboxamide | A |
| Carbendazim | 0.10 | 0.01 | - | Fungicide | Benzimidazole | NA |
| **Chlormequat** | **7.00** | 0.01 | **25** | Plant growth regulator | Quarternary ammonium compound | A |
| Chlorpropham | 0.01 | 0.02** | - | Herbicide, Plant growth regulator | Carbamate | A |
| Chlorpyrifos | 0.50 | 0.01 | - | Insecticide, Acaricide | Organophosphate | A |
| Chlorpyrifos-methyl | **0.05** | 0.01 | - | Insecticide, Acaricide | Organophosphate | A |
| Clothianidin | 0.02 | 0.01 | - | Insecticide | Neonicotinoid | NA |
| Cyfluthrin | 0.04 | 0.01 | - | Insecticide, Acaricide | Pyrethroid | NA |
| Cyhalothrin-lambda | 0.05 | 0.01 | - | Insecticide | Pyrethroid | NA |
| **Cypermethrin** | **2.00** | 0.01 | **15** | Insecticide, Acaricide | Pyrethroid | A |
| Cyproconazole | 0.10 | 0.01 | - | Fungicide | Triazole | A |
| Cyprodinil | 0.50 | 0.01 | - | Fungicide | Anilinopyrimidine | A |
| DDT | 0.05 | 0.01 | - | Insecticide | Organochlorine | NA |
| **Deltamethrin** | **1.00** | 0.01 | **5** | Insecticide | Pyrethroid | A |
| * LOD, limit of detection. **, the LOD was assumed to be the same as the limit of quantification (LOQ; the lowest concentration at which the analyte can not only be reliably detected but also reliably quantified) defined by laboratory carrying out the analysis. **^a^**, European Commission (2018) <https://ec.europa.eu/food/plant/pesticides/eu-pesticides-database/public/?event=activesubstance.selection&language=EN>; A, Approved for use in the EU; NA, not approved for use in the EU; ND, MRL not defined by the EU because it is not included in the EU pesticide database or is not a plant protection product; **^b^**, PPDB (2019) <https://sitem.herts.ac.uk/aeru/ppdb/en/atoz.htm>  **^1^** Not a permitted food additive, allowed as a post-harvest treatment (only in 4 EU countries). **^2^** Only use in greenhouses with a permanent structure may be authorised in the EU. | | | | | | |

| ***Table S3 cont.*** List of pesticide compounds analysed in 2016 and their EU-maximum residue limit (MRL), limit of detection (LOD) in the analytical tests, product type, chemical group and approval status in the EU | | | | | | |
| --- | --- | --- | --- | --- | --- | --- |
|  | **EU MRL for** | **LOD*** | **No. of** |  |  | **Approval status** |
| **Compound** | **Wheat ^a^** | **(mg/kg)** | **positive samples** | **Type of product ^b^** | **Chemical Group ^b^** | **in the EU ^a^** |
| Diazinon | 0.01 | 0.01 | - | Insecticide, Acaricide | Organophosphate | NA |
| Difenoconazole | 0.10 | 0.01 | - | Fungicide | Triazole | A |
| Dimethoate | 0.05 | 0.01 | - | Insecticide, Acaricide | Organophosphate | A |
| Epoxiconazole | 0.60 | 0.01 | - | Fungicide | Triazole | A |
| Fenhexamid | 0.01 | 0.01 | - | Fungicide | Fenhexamid | A |
| Fenpropimorph | 0.15 | 0.01 | - | Fungicide | Morpholine | A |
| Fenvalerate | 0.20 | 0.01 | - | Insecticide, Acaricide | Pyrazolium | NA |
| Fluazifop (free acid) | 0.01 | 0.05** | - | Herbicide | Unclassified | NA |
| Fludioxonil | 0.01 | 0.01 | - | Fungicide | Phenylpyrrole | A |
| Flutriafol | 0.15 | 0.01 | - | Fungicide | Triazole | A |
| Folpet | 0.40 | 0.01 | - | Fungicide | Phthalimide | A |
| Glyphosate | 10.0 | 3.00** | - | Herbicide | Phosphonoglycine | A |
| Imazalil | 0.05 | 0.01 | - | Fungicide | Imidazole | A |
| Imidacloprid | 0.10 | 0.01 | - | Insecticide | Neonicotinoid | A |
| Malathion | 8.00 | 0.01 | - | Insecticide, Acaricide | Organophosphate | A |
| Mepiquat | 3.00 | 0.01 | **3** | Plant growth regulator | Quarternary ammonium compound | A |
| Metalaxyl | 0.01 | 0.01 | - | Fungicide | Phenylamide | A |
| Metribuzin | 0.10 | 0.02** | - | Herbicide | Triazinone | A |
| Omethoate | 0.01 | 0.01 | - | Insecticide, Acaricide | Organophosphate | NA |
| * LOD, limit of detection. **, the LOD was assumed to be the same as the limit of quantification (LOQ; the lowest concentration at which the analyte can not only be reliably detected but also reliably quantified) defined by laboratory carrying out the analysis. **^a^**, European Commission (2018) <https://ec.europa.eu/food/plant/pesticides/eu-pesticides-database/public/?event=activesubstance.selection&language=EN>; A, Approved for use in the EU; NA, not approved for use in the EU; ND, MRL not defined by the EU because it is not included in the EU pesticide database or is not a plant protection product; **^b^**, PPDB (2019) <https://sitem.herts.ac.uk/aeru/ppdb/en/atoz.htm> | | | | | | |

| ***Table S3 cont.*** List of pesticide compounds analysed in 2016 and their EU-maximum residue limit (MRL), limit of detection (LOD) in the analytical tests, product type, chemical group and approval status in the EU | | | | | | |
| --- | --- | --- | --- | --- | --- | --- |
|  | **EU MRL for** | **LOD*** | **No. of** |  |  | **Approval status** |
| **Compound** | **Wheat ^a^** | **(mg/kg)** | **positive samples** | **Type of product ^b^** | **Chemical Group ^b^** | **in the EU ^a^** |
| Pendimethalin | **0.05** | 0.01 | **3** | Herbicide | Dinitroaniline | A |
| Piperonyl butoxide | **ND** | 0.01 | **31** | Not a plant protection product | Cyclic aromatic | NA |
| Pirimicarb | **0.05** | 0.01 | - | Insecticide | Carbamate | A |
| Pirimicarb-desmethyl | **0.05** | 0.01 | - | Insecticide | Carbamate | NA |
| Pirimiphos-ethyl | **0.01 ^1^** | 0.01 | - | Insecticide | Organophosphate | NA |
| Pirimiphos-methyl | **5.00** | 0.01 | **3** | Insecticide | Organophosphate | A |
| Propiconazole | 0.09 | 0.01 | - | Fungicide | Triazole | NA |
| Pyraclostrobin | 0.20 | 0.01 | - | Fungicide, Plant growth regulator | Strobilurin | A |
| Pyrethrins | **3.00** | 0.01 | - | Insecticide | Plant derived extract | A |
| Pyrimethanil | 0.05 | 0.01 | - | Fungicide | Anilinopyrimidine | A |
| Spinosad | **2.00** | 0.01 | - | Insecticide | Micro-organism derived | A |
| Spiroxamine | 0.05 | 0.01 | - | Fungicide | Morpholine | A |
| Tau-Fluvalinate | 0.05 | 0.01 | - | Insecticide | Synthetic pyrethroid | A |
| Tebuconazole | **0.30** | 0.01 | **2** | Fungicide | Triazole | A |
| Tefluthrin | **0.05** | 0.02** | - | Insecticide | Pyrethroid | A |
| Thiacloprid | 0.10 | 0.01 | - | Insecticide | Neonicotinoid | A |
| Thiamethoxam | 0.05 | 0.01 | - | Insecticide | Neonicotinoid | A |
| Thiophanate-methyl | 0.05 | 0.01 | - | Fungicide | Benzimidazole | A |
| Triadimenol | 0.10 | 0.01 | - | Fungicide | Triazole | A |
| * LOD, limit of detection. **, the LOD was assumed to be the same as the limit of quantification (LOQ; the lowest concentration at which the analyte can not only be reliably detected but also reliably quantified) defined by laboratory carrying out the analysis. **^a^**, European Commission (2018) <https://ec.europa.eu/food/plant/pesticides/eu-pesticides-database/public/?event=activesubstance.selection&language=EN>; A, Approved for use in the EU; NA, not approved for use in the EU; ND, MRL not defined by the EU because it is not included in the EU pesticide database or is not a plant protection product; **^b^**, PPDB (2019) <https://sitem.herts.ac.uk/aeru/ppdb/en/atoz.htm> **^1^**, Default MRL of 0.01 mg/kg according to Art 18(1)(b) Reg 396 / 2005. | | | | | | |

| ***Table S4.*** List of pesticide compounds analysed in **2017** and their EU-maximum residue limit (MRL), limit of detection (LOD) in the analytical tests, product type, chemical group and approval status in the EU | | | | | | |
| --- | --- | --- | --- | --- | --- | --- |
|  | **EU MRL** |  | **No. of** |  |  | **Approval** |
| **Compound** | **for**  **Wheat ^a^** | **LOD***  **(mg/kg)** | **positive samples** | **Type of product ^b^** | **Chemical Group ^b^** | **status**  **in the EU ^a^** |
| 1,4-Dimethylnapthalene | ND | 0.01 | - | Plant growth regulator |  | ND |
| 2-(1-Naphthyl)acetamide | 0.06 | 0.01 | - | Plant growth regulator | Auxin | A |
| 2,4,6-Trichlorophenol | 0.50 **^1^** | 0.01 | - | Fungicide,Herbicide | Unclassified | NA |
| 2-Methyl-4,6-dinitrophenol | ND | 0.01 | - |  |  | ND |
| **2-phenylphenol** | **0.02** | 0.01 | **12** | Fungicide | Phenol | A**^3^** |
| 3-hydroxycarbofuran | ND | 0.01 | - |  |  | ND |
| 4,4-Dichlorobenzophenone | ND | 0.01 | - |  |  | ND |
| 6-Benzyladenine | 0.01 **^2^** | 0.01 | - | Plant growth regulator |  | A |
| 9,10-Anthraquinone | 0.01 | 0.01 | - | Repellent | Unclassified | NA |
| Abamectin | 0.01 | 0.01 | - | Insecticide,Acaricide |  | A |
| Acephate | 0.01 | 0.01 | - | Insecticide | Organophosphate | NA |
| Acetamiprid | 0.10 | 0.01 | - | Insecticide | chloropyridinylneonicotinoids | A |
| Acetochlor | 0.01 | 0.01 | - | Herbicide | Chloroacetamide | NA |
| Acibenzolar-S-methyl | 0.05 | 0.01 | - | Plantactivator | Benzothiadiazole | A |
| Aclonifen | 0.01 | 0.01 | - | Herbicide | Diphenylether | A |
| Acrinathrin | 0.01 | 0.01 | - | Acaricide | Pyrethroid | A |
| Alachlor | 0.01 | 0.01 | - | Herbicide | Chloroacetamide | NA |
| Aldicarb | 0.02 | 0.01 | - | Insecticide,Acaricide,Nematicide | Carbamate | NA |
| * LOD, limit of detection.  **^a^**, European Commission (2018) <https://ec.europa.eu/food/plant/pesticides/eu-pesticides-database/public/?event=activesubstance.selection&language=EN>; A, Approved for use in the EU; NA, not approved for use in the EU; ND, MRL not defined by the EU because it is not included in the EU pesticide database or is not a plant protection product; **^b^**, PPDB (2019) <https://sitem.herts.ac.uk/aeru/ppdb/en/atoz.htm>  **^1^** MRL for prochloraz is for the sum of prochloraz and its metabolites containing the 2,4,6-Trichlorophenol moiety expressed as prochloraz) **^2^** Default MRL of 0.01 mg/kg according to Art 18(1)(b) Reg 396 / 2005.  **^3^** Not a permitted food additive, allowed as a post-harvest treatment (only in 4 EU countries). | | | | | | |

| ***Table S4 cont.*** List of pesticide compounds analysed in **2017** and their EU-maximum residue limit (MRL), limit of detection (LOD) in the analytical tests, product type, chemical group and approval status in the EU | | | | | | |
| --- | --- | --- | --- | --- | --- | --- |
|  | **EU MRL for** | **LOD*** | **No. of** |  |  | **Approval status** |
| **Compound** | **Wheat ^a^** | **(mg/kg)** | **positive samples** | **Type of product ^b^** | **Chemical Group ^b^** | **in the EU ^a^** |
| Aldicarb sulphone **^1^** | 0.02 ^1^ | 0.01 | - | Insecticide,Acaricide,Nematicide | Carbamate | NA |
| Aldicarb sulphoxide **^1^** | 0.02 ^1^ | 0.01 | - | Insecticide,Acaricide,Nematicide | Carbamate | NA |
| Aldrin | 0.01 | 0.01 | - | Insecticide | Organochlorine | NA |
| Ametryn | 0.01 ^2^ | 0.01 | - | Herbicide | Triazine | NA |
| Aminocarb | ND | 0.01 | - | Insecticide | Carbamate | ND |
| Amitraz | 0.05 | 0.01 | - | Insecticide,Acaricide | Amidine | NA |
| Atraton | ND | 0.01 | - | Herbicide | Methoxytriazine | ND |
| Atrazine | 0.05 | 0.01 | - | Herbicide | Triazine | NA |
| Azaconazole | 0.01 **^2^** | 0.01 | - | Fungicide,Insecticide | Triazole | NA |
| Azadirachtin | 1.00 | 0.01 | - | Insecticide | Plant extract | A |
| Azinphos ethyl | 0.05 | 0.01 | - | Insecticide,Acaricide | Organophosphate | NA |
| Azinphos methyl | 0.05 | 0.01 | - | Insecticide,Acaricide | Organophosphate | NA |
| Azobenzene | ND | 0.01 | - | Acaricide,Ovicide,Miticide | Bridgeddiphenyl | ND |
| Azoxystrobin | 0.50 | 0.01 | - | Fungicide | Strobilurin | A |
| Benalaxyl | 0.05 | 0.01 | - | Fungicide | Acylaminoacid | A |
| Bendiocarb | ND | 0.01 | - | Insecticide | Carbamate | ND |
| Bifenthrin | 0.50 | 0.01 | - | Herbicide | Dinitroaniline | A |
| Benfuracarb | 0.01 | 0.01 | - | Insecticide,Nematicide | Carbamate | NA |
| * LOD, limit of detection.  **^a^**, European Commission (2018) <https://ec.europa.eu/food/plant/pesticides/eu-pesticides-database/public/?event=activesubstance.selection&language=EN>; A, Approved for use in the EU; NA, not approved for use in the EU; ND, MRL not defined by the EU because it is not included in the EU pesticide database or is not a plant protection product; **^b^**, PPDB (2019) <https://sitem.herts.ac.uk/aeru/ppdb/en/atoz.htm> ^1^ MRL for Aldicarb (sum of aldicarb, its sulfoxide and its sulfone, expressed as aldicarb) **^2^** Default MRL of 0.01 mg/kg according to Art 18(1)(b) Reg 396 / 2005. | | | | | | |

| ***Table S4 cont.*** List of pesticide compounds analysed in **2017** and their EU-maximum residue limit (MRL), limit of detection (LOD) in the analytical tests, product type, chemical group and approval status in the EU | | | | | | |
| --- | --- | --- | --- | --- | --- | --- |
|  | **EU MRL for** | **LOD*** | **No. of** |  |  | **Approval status** |
| **Compound** | **Wheat ^a^** | **(mg/kg)** | **positive samples** | **Type of product ^b^** | **Chemical Group ^b^** | **in the EU ^a^** |
| Benthiavalicarb-isopropyl | 0.02 **^1^** | 0.01 | - | Fungicide | Carbamate | A |
| Bifenazate | 0.02 | 0.01 | - | Insecticide,Acaricide | Hydrazinecarboxylate | A |
| Bifenox | 0.02 | 0.01 | - | Herbicide | Diphenylether | A |
| Bifenthrin | 0.50 | 0.01 | - | Insecticide,Acaricide | Pyrethroid | A**^2^** |
| Binapacryl | 0.02 | 0.01 | - | Fungicide,Insecticide,Miticide | Dinitrophenol | NA |
| Biphenyl | 0.01 | 0.05 | - | Fungicide | Aromatichydrocarbon | NA |
| Bitertanol | 0.01 | 0.01 | - | Fungicide | Triazole | NA |
| Boscalid | 0.80 | 0.01 | - | Fungicide | Carboxamide | A |
| Bromacil | ND | 0.01 | - | Herbicide | Uracil | ND |
| Bromophos | 0.01 | 0.01 | - | Insecticide | Organophosphate | NA |
| Bromophos-Ethyl | 0.01 | 0.01 | - | Insecticide | Organophosphate | NA |
| Bromopropylate | 0.01 | 0.01 | - | Acaricide | Benzilate | NA |
| Bromuconazole | 0.20 | 0.01 | - | Fungicide | Triazole | A |
| Bupirimate | 0.05 | 0.01 | - | Fungicide | Pyrimidinol | A |
| Buprofezine | ND | 0.01 | - | Insecticide,Acaricide | Unclassified | ND |
| Butachlor | 0.01 **^3^** | 0.01 | - | Herbicide | Chloroacetamide | NA |
| Butocarboxim | 0.01 **^3^** | 0.01 | - | Insecticide | Butocarboxim | NA |
| Butoxycarboxim | 0.01 **^3^** | 0.01 | - | Insecticide,Acaricide | Carbamate | NA |
| * LOD, limit of detection.  **^a^**, European Commission (2018) <https://ec.europa.eu/food/plant/pesticides/eu-pesticides-database/public/?event=activesubstance.selection&language=EN>; A, Approved for use in the EU; NA, not approved for use in the EU; ND, MRL not defined by the EU because it is not included in the EU pesticide database or is not a plant protection product; **^b^**, PPDB (2019) <https://sitem.herts.ac.uk/aeru/ppdb/en/atoz.htm>  **^1^** MRL for Benthiavalicarb (Benthiavalicarb-isopropyl and its enantiomer and its diastereomers, expressed as benthiavalicarb-isopropyl) **^2^** Only uses as insecticide in greenhouses with a permanent structure may be authorised. **^3^** Default MRL of 0.01 mg/kg according to Art 18(1)(b) Reg 396 / 2005. | | | | | | |

| ***Table S4 cont.*** List of pesticide compounds analysed in **2017** and their EU-maximum residue limit (MRL), limit of detection (LOD) in the analytical tests, product type, chemical group and approval status in the EU | | | | | | |
| --- | --- | --- | --- | --- | --- | --- |
|  | **EU MRL for** | **LOD*** | **No. of** |  |  | **Approval status** |
| **Compound** | **for wheat ^a^** | **(mg/kg)** | **positive samples** | **Type of product ^b^** | **Chemical Group ^b^** | **in the EU ^a^** |
| Butralin | 0.01 | 0.01 | - | Herbicide,Plant growth regulator | Dinitroaniline | NA |
| Cadusafos | 0.01 | 0.01 | - | Insecticide,Nematicide | Organophosphate | NA |
| Captan | 0.07 | 0.01 | - | Fungicide | Phthalimide | A |
| Carbaryl | 0.50 | 0.01 | - | Insecticide,Plant growth regulator | Carbamate | NA |
| Carbendazim | 0.10 | 0.01 | - | Fungicide | Benzimidazole | NA |
| Carbetamide | 0.01 | 0.01 | - | Herbicide | Carbamate | A |
| Carbofuran | 0.01 | 0.01 | - | Insecticide,Acaricide,Nematicide | Carbamate | NA |
| Carbophenothion | 0.01 **^1^** | 0.01 | - | Insecticide,Acaricide | Organophosphate | NA |
| Carboxine | 0.03 | 0.01 | - | Fungicide | Oxathiin | A |
| Carfentrazone Ethyl | 0.05 | 0.01 | - | Herbicide |  | A |
| Carpropamid | 0.01 **^1^** | 0.01 | - | Fungicide | Cyclopropanecarboxamide | NA |
| Chinomethionat | 0.01 **^1^** | 0.01 | - | Fungicide,Acaricide | Carbamate | NA |
| Chlorantraniliprole | 0.02 | 0.01 | - | Insecticide | Anthranilicdiamide | A |
| Chlorbenzilate | ND | 0.01 | - |  |  | ND |
| Chlorbromuron | 0.01 **^1^** | 0.01 | - | Herbicide | Urea | NA |
| Chlorbufam | 0.01 | 0.01 | - | Herbicide | Carbanilate | NA |
| Chlordane | 0.01 **^1^** | 0.01 | - | NA | Organochlorine | NA |
| Chlordimeform | ND | 0.01 | - | Insecticide,Acaricide | Formamidine | ND |
| * LOD, limit of detection.  **^a^**, European Commission (2018) <https://ec.europa.eu/food/plant/pesticides/eu-pesticides-database/public/?event=activesubstance.selection&language=EN>; A, Approved for use in the EU; NA, not approved for use in the EU; ND, MRL not defined by the EU because it is not included in the EU pesticide database or is not a plant protection product; **^b^**, PPDB (2019) <https://sitem.herts.ac.uk/aeru/ppdb/en/atoz.htm>  **^1,^** Default MRL of 0.01 mg/kg according to Art 18(1)(b) Reg 396 / 2005. | | | | | | |

| ***Table S4 cont.*** List of pesticide compounds analysed in **2017** and their EU-maximum residue limit (MRL), limit of detection (LOD) in the analytical tests, product type, chemical group and approval status in the EU | | | | | | |
| --- | --- | --- | --- | --- | --- | --- |
|  | **EU MRL** |  | **No. of** |  |  |  |
| **Compound** | **for wheat ^a^** | **LOD***  **(mg/kg)** | **positive samples** | **Type of product ^b^** | **Chemical Group ^b^** | **Approval status**  **in the EU ^a^** |
| Chlorethoxyfos | 0.01 **^1^** | 0.01 | - | Insecticide | Organophosphate | NA |
| Chlorfenapyr | 0.02 | 0.01 | - | Insecticide,Acaricide | Pyrrole | NA |
| Chlorfenson | 0.01 | 0.01 | - | Insecticide,Acaricide | Bridgeddiphenyl | NA |
| Chlorfenvinphos | 0.01 | 0.01 | - | Insecticide | Organophosphate | NA |
| Chlorfluazuron | 0.01 **^1^** | 0.01 | - | Insecticide | Benzoylurea | NA |
| Chloridazon | 0.10 | 0.01 | - | Herbicide | Pyridazinone | NA |
| Chlormephos | 0.01 **^1^** | 0.01 | - | Insecticide | Organophosphate | NA |
| **Chlormequat** | 7.00 | 0.01 | **66** | Plant growth regulator | Quarternaryammoniumcompound | A |
| Chloropropylate | 0.01 **^1^** | 0.01 | - | Acaricide | Bridgeddiphenyl | NA |
| Chlorothalonil | 0.10 | 0.01 | - | Fungicide | Chloronitrile | A |
| Chlorotoluron | 0.10 | 0.01 | - | Herbicide | Urea | A |
| Chlorpropham | 0.10 | 0.01 | - | Herbicide,Plant growth regulator | Carbamate | A |
| Chlorpyrifos | 0.50 | 0.01 | - | Insecticide,Acaricide | Organophosphate | A |
| Chlorpyrifos methyl | **0.05** | 0.01 | **1** | Insecticide,Acaricide | Organophosphate | A |
| Chlorthal Dimethyl | 0.01 | 0.01 | - | Herbicide |  | NA |
| Chlorthion | ND | 0.01 | - | Insecticide | Organophosphate | ND |
| Chlorthiophos | 0.01 **^1^** | 0.01 | - | Insecticide | Organophosphate | NA |
| Chlozolinate | 0.01 | 0.01 | - | Fungicide | Oxazolidin | NA |
| * LOD, limit of detection.  **^a^**, European Commission (2018) <https://ec.europa.eu/food/plant/pesticides/eu-pesticides-database/public/?event=activesubstance.selection&language=EN>; A, Approved for use in the EU; NA, not approved for use in the EU; ND, MRL not defined by the EU because it is not included in the EU pesticide database or is not a plant protection product; **^b^**, PPDB (2019) <https://sitem.herts.ac.uk/aeru/ppdb/en/atoz.htm> **^1^**, Default MRL of 0.01 mg/kg according to Art 18(1)(b) Reg 396 / 2005. | | | | | | |

| ***Table S4 cont.*** List of pesticide compounds analysed in **2017** and their EU-maximum residue limit (MRL), limit of detection (LOD) in the analytical tests, product type, chemical group and approval status in the EU | | | | | | |
| --- | --- | --- | --- | --- | --- | --- |
|  |  |  | **No. of** |  |  | **Approval** |
| **Compound** | **EU MRL**  **for wheat ^a^** | **LOD***  **(mg/kg)** | **positive samples** | **Type of product ^b^** | **Chemical Group ^b^** | **status**  **in the EU ^a^** |
| cis-1,2,3,6-Tetrahydrophthalimide | ND | 0.01 | - | Metabolite | Unclassified | ND |
| Clodinafop propargy | 0.02 | 0.01 | - | Herbicide | Aryloxyphenoxypropionate | NA |
| Clofentezine | 0.02 | 0.01 | - | Acaricide | Tetrazine | A |
| Clomazone | 0.01 | 0.01 | - | Herbicide | Isoxazolidinone | A |
| Cuintocet mexyl | ND | 0.01 | - | Not a plant protection product | Unclassified | ND |
| Clothianidin | 0.02 | 0.01 | - | Insecticide | Neonicotinoid | NA |
| Coumaphos | 0.01 **^1^** | 0.01 | - | Insecticide |  | NA |
| Cyanazine | 0.01 **^1^** | 0.01 | - | Herbicide | Triazine | NA |
| Cyazofamid | 0.02 | 0.01 | - | Fungicide | Cyanoimidazole | A |
| Cycluron | 0.01 **^1^** | 0.01 | - | Herbicide | Urea | NA |
| Cyflufenamid | 0.05 | 0.01 | - | Fungicide | Amidoxine | A |
| Cyfluthrin | 0.04 | 0.01 | - | Insecticide,Acaricide | Pyrethroid | NA |
| Cymoxanil | 0.01 | 0.01 | - | Fungicide | Cyanoacetamideoxime | A |
| Cypermethrin | 2.00 | 0.01 | - | Insecticide,Acaricide | Pyrethroid | A |
| Cyphenothrin | ND | 0.01 |  | Insecticide | Pyrethroid | ND |
| Cyproconazole | 0.10 | 0.01 | - | Fungicide | Triazole | A |
| Cyprodinil | 0.50 | 0.01 | - | Fungicide | Anilinopyrimidine | A |
| Cyromazine | 0.05 | 0.01 | - | Insecticide | Triazine | A |
| * LOD, limit of detection.  **^a^**, European Commission (2018) <https://ec.europa.eu/food/plant/pesticides/eu-pesticides-database/public/?event=activesubstance.selection&language=EN>; A, Approved for use in the EU; NA, not approved for use in the EU; ND, MRL not defined by the EU because it is not included in the EU pesticide database or is not a plant protection product; **^b^**, PPDB (2019) <https://sitem.herts.ac.uk/aeru/ppdb/en/atoz.htm> **^1^**, Default MRL of 0.01 mg/kg according to Art 18(1)(b) Reg 396 / 2005. | | | | | | |

| ***Table S4 cont.*** List of pesticide compounds analysed in **2017** and their EU-maximum residue limit (MRL), limit of detection (LOD) in the analytical tests, product type, chemical group and approval status in the EU | | | | | | |
| --- | --- | --- | --- | --- | --- | --- |
|  | **EU MRL for** | **LOD*** | **No. of** |  |  | **Approval status** |
| **Compound** | **Wheat ^a^** | **(mg/kg)** | **positive samples** | **Type of product ^b^** | **Chemical Group ^b^** | **in the EU ^a^** |
| Cythioate | ND | 0.01 | - |  |  | ND |
| DEET | ND | 0.01 | - | Insect repellent |  | ND |
| **Deltamethrin** | **1.00** | **0.01** | **5** | Insecticide | Pyrethroid | A |
| Demeton | 0.02 | 0.01 | - | Insecticide,Acaricide | Organophosphate | NA |
| Demeton-s-methyl | 0.02 | 0.01 | - | Insecticide,Acaricide | Organophosphate | NA |
| Demeton-s-methyl sulphone | 0.02 | 0.01 | - | Insecticide | Organophosphate | NA |
| Desmedipham | 0.01 | 0.01 | - | Herbicide | Carbamate | A |
| Desmetryn | 0.01 **^1^** | 0.01 | - | Herbicide | Methylthiotriazine | NA |
| Diafenthiuron | 0.01 **^1^** | 0.01 | - | Insecticide,Acaricide | Thiourea | NA |
| Dialifos | 0.01 **^1^** | 0.01 | - | Insecticide,Acaricide | Organophosphate | NA |
| Diazinon | 0.01 | 0.01 | - | Insecticide,Acaricide | Organophosphate | NA |
| Dichlobenil | 0.01 | 0.01 | - | Herbicide | Benzonitrile | NA |
| Dichlofenthion | 0.01 **^1^** | 0.01 | - | Insecticide | Organophosphate | NA |
| Dichlofluanid | 0.01 **^1^** | 0.01 | - | Fungicide | Sulphamide | NA |
| Dichlorvos | 0.01 | 0.01 | - | Insecticide,Acaricide | Organophosphate | NA |
| Diclobutrazol | 0.01 **^1^** | 0.01 | - | Fungicide | Conazole | NA |
| Dicloran | 0.02 | 0.01 | - | Fungicide | Chlorophenyl | NA |
| Dicofol | 0.02 | 0.01 | - | Acaricide | Organochlorine | NA |
| * LOD, limit of detection.  **^a^**, European Commission (2018) <https://ec.europa.eu/food/plant/pesticides/eu-pesticides-database/public/?event=activesubstance.selection&language=EN>; A, Approved for use in the EU; NA, not approved for use in the EU; ND, MRL not defined by the EU becasue it is not included in the EU pesticide database or is not a plant protetion product; **^b^**, PPDB (2019) <https://sitem.herts.ac.uk/aeru/ppdb/en/atoz.htm>  ^1^, Default MRL of 0.01 mg/kg according to Art 18(1)(b) Reg 396 / 2005. | | | | | | |

| ***Table S4 cont.*** List of pesticide compounds analysed in **2017** and their EU-maximum residue limit (MRL), limit of detection (LOD) in the analytical tests, product type, chemical group and approval status in the EU | | | | | | |
| --- | --- | --- | --- | --- | --- | --- |
|  | **EU MRL for** | **LOD*** | **No. of** |  |  | **Approval status** |
| **Compound** | **Wheat ^a^** | **(mg/kg)** | **positive samples** | **Type of product ^b^** | **Chemical Group ^b^** | **in the EU ^a^** |
| Dicrotophos | 0.01 **^1^** | 0.01 | - | Insecticide,Acaricide | Organophosphate | NA |
| Dieldrin | 0.01 | 0.01 | - | Insecticide | Chlorinatedhydrocarbon | NA |
| Diethofencarb | 0.01 | 0.01 | - | Fungicide | Carbamate | A |
| Difenoconazole | 0.10 | 0.01 | - | Fungicide | Triazole | A |
| Diflubenzuron | 0.01 | 0.01 | - | Insecticide | Benzoylurea | A |
| Diflufenican | 0.02 | 0.01 | - | Herbicide | Carboxamide | A |
| Dimefuron | 0.01 **^1^** | 0.01 | - | Herbicide | Oxadiazolone/phenylurea | NA |
| Dimethenamid | 0.01 | 0.01 | - | Herbicide | Chloroacetamide | NA |
| Dimethoate | 0.05 | 0.01 | - | Insecticide,Acaricide | Organophosphate | A |
| Dimethomorph | 0.01 | 0.01 | - | Fungicide | Morpholine | A |
| Dimoxystrobin | 0.08 | 0.01 | - | Fungicide | Strobilurin | A |
| Diniconazole | 0.01 | 0.01 | - | Fungicide | Triazole | NA |
| Dinotefuran | 0.01 **^1^** | 0.01 | - | Insecticide | Neonicotinoid | NA |
| Dinoterb | 0.01 | 0.01 | - | Herbicide | Dinitrophenol | NA |
| Dioxabenzofos | ND | 0.01 | - | Insecticide | Organophosphate | ND |
| Dioxacarb | 0.01 **^1^** | 0.01 | - | Insecticide | Carbamate | NA |
| Diphenamid | 0.01 **^1^** | 0.01 | - | Herbicide | Alkanamide | NA |
| Diphenylamine | 0.05 | 0.01 | - | Plant growth regulator | Amine | NA |
| * LOD, limit of detection.  **^a^**, European Commission (2018) <https://ec.europa.eu/food/plant/pesticides/eu-pesticides-database/public/?event=activesubstance.selection&language=EN>; A, Approved for use in the EU; NA, not approved for use in the EU; ND, MRL not defined by the EU because it is not included in the EU pesticide database or is not a plant protection product; **^b^**, PPDB (2019) <https://sitem.herts.ac.uk/aeru/ppdb/en/atoz.htm>  ^1^, Default MRL of 0.01 mg/kg according to Art 18(1)(b) Reg 396 / 2005 | | | | | | |

| ***Table S4 cont.*** List of pesticide compounds analysed in **2017** and their EU-maximum residue limit (MRL), limit of detection (LOD) in the analytical tests, product type, chemical group and approval status in the EU | | | | | | |
| --- | --- | --- | --- | --- | --- | --- |
|  | **EU MRL for** | **LOD*** | **No. of** |  |  | **Approval status** |
| **Compound** | **Wheat ^a^** | **(mg/kg)** | **positive samples** | **Type of product ^b^** | **Chemical Group ^b^** | **in the EU ^a^** |
| Disulfoton | 0.02 | 0.01 | - | Insecticide | Organophosphate | NA |
| Disulfoton sulfoxide | 0.02 **^1^** | 0.01 | - | Metabolite | Organophosphate | NA |
| Disulfoton sulphone | 0.02 **^1^** | 0.01 | - |  |  | NA |
| Ditalimfos | 0.01 **^2^** | 0.01 | - | Fungicide | Organophosphate | NA |
| Diuron | 0.01 | 0.01 | - | Herbicide | Phenylamide | A |
| DMSA | ND | 0.01 | - |  |  | ND |
| DMST | ND | 0.01 | - |  |  | ND |
| Dodemorph | 0.01 | 0.01 | - | Fungicide | Morpholine | A |
| Dodine | 0.01 | 0.01 | - | Fungicide | Guanidine | A |
| Edifenphos | 0.01 **^2^** | 0.01 | - | Fungicide | Organophosphate | NA |
| Emamectin | 0.01 | 0.01 | - | Insecticide |  | A |
| Endosulphan alpha | ND | 0.01 | - | Insecticide,Acaricide | Organochlorine | ND |
| Endosulphan beta | ND | 0.01 | - | Insecticide,Acaricide | Organochlorine | ND |
| Endosulphan sulphate | ND | 0.01 | - |  |  | ND |
| Endrin | 0.01 | 0.01 | - | Insecticide,Avicide | Organochlorine | NA |
| Epn | 0.01 **^2^** | 0.01 | - | Insecticide,Acaricide | Organophosphate | NA |
| Epoxiconazole | 0.60 | 0.01 | - | Fungicide | Triazole | A |
| EPTC | 0.01 | 0.01 | - | Herbicide | Thiocarbamate | NA |
| * LOD, limit of detection.  **^a^**, European Commission (2018) <https://ec.europa.eu/food/plant/pesticides/eu-pesticides-database/public/?event=activesubstance.selection&language=EN>; A, Approved for use in the EU; NA, not approved for use in the EU; ND, MRL not defined by the EU because it is not included in the EU pesticide database or is not a plant protection product; **^b^**, PPDB (2019) <https://sitem.herts.ac.uk/aeru/ppdb/en/atoz.htm>  ^1^ MRL for disulfoton (sum of disulfoton, disulfoton sulfoxide and disulfoton sulfone)  **^2^**, Default MRL of 0.01 mg/kg according to Art 18(1)(b) Reg 396 / 2005. | | | | | | |

| ***Table S4 cont.*** List of pesticide compounds analysed in **2017** and their EU-maximum residue limit (MRL), limit of detection (LOD) in the analytical tests, product type, chemical group and approval status in the EU | | | | | | |
| --- | --- | --- | --- | --- | --- | --- |
|  | **EU MRL for** | **LOD*** | **No. of** |  |  | **Approval status** |
| **Compound** | **Wheat ^a^** | **(mg/kg)** | **positive samples** | **Type of product ^b^** | **Chemical Group ^b^** | **in the EU ^a^** |
| Etaconazole | 0.01 **^1^** | 0.01 | - | Fungicide | Conazole | NA |
| Ethidimuron | 0.01 **^1^** | 0.01 | - | Herbicide | Thiadiazolylurea | NA |
| Ethiofencarb | 0.01 **^1^** | 0.01 | - | Insecticide | Carbamate | NA |
| Ethiofencarb sulfone | ND | 0.01 | - | Metabolite | Unclassified | ND |
| Ethiofencarb sulfoxide | ND | 0.01 | - | Metabolite | Unclassified | ND |
| Ethion | 0.01 | 0.01 | - | Insecticide,Acaricide | Organophosphate | NA |
| Ethiprole | 0.01 **^1^** | 0.01 | - | Insecticide | Phenylpyrazole | NA |
| Ethirimol | 0.05 | 0.01 | - | Fungicide | Pyrimidinol | NA |
| Ethofumesate | 0.03 | 0.01 | - | Herbicide | Benzofuran | A |
| Ethoprophos | 0.02 | 0.01 | - | Insecticide,Nematicide | Organophosphate | NA |
| **Ethoxyquin** | **0.05** | **0.05** | - | Plant growth regulator | Quinoline | NA |
| Etofenprox | 0.01 | 0.01 | - | Insecticide | Pyrethroid | A |
| Etoxazole | 0.01 | 0.01 | - | Insecticide | Diphenyloxazoline | A |
| Etridiazole | 0.05 | 0.01 | - | Fungicide | Aromatichydrocarbon | A |
| Etrimfos | 0.01 **^1^** | 0.01 | - | Insecticide,Acaricide | Organophosphate | NA |
| **Famoxadone** | **0.10** | **0.05** | - | Fungicide | Oxazole | A |
| Famphur | ND | 0.01 | - |  |  | ND |
| Fenamidone | 0.01 | 0.01 | - | Fungicide | Imidazole | NA |
| * LOD, limit of detection.  **^a^**, European Commission (2018) <https://ec.europa.eu/food/plant/pesticides/eu-pesticides-database/public/?event=activesubstance.selection&language=EN>; A, Approved for use in the EU; NA, not approved for use in the EU; ND, MRL not defined by the EU because it is not included in the EU pesticide database or is not a plant protection product; **^b^**, PPDB (2019) <https://sitem.herts.ac.uk/aeru/ppdb/en/atoz.htm> **^1^**, Default MRL of 0.01 mg/kg according to Art 18(1)(b) Reg 396 / 2005. | | | | | | |

| ***Table S4 cont.*** List of pesticide compounds analysed in **2017** and their EU-maximum residue limit (MRL), limit of detection (LOD) in the analytical tests, product type, chemical group and approval status in the EU | | | | | | |
| --- | --- | --- | --- | --- | --- | --- |
|  | **EU MRL for** | **LOD*** | **No. of** |  |  | **Approval status** |
| **Compound** | **Wheat ^a^** | **(mg/kg)** | **positive samples** | **Type of product ^b^** | **Chemical Group ^b^** | **in the EU ^a^** |
| Fenamiphos | 0.02 **^1^** | 0.01 | - | Nematicide | Organophosphate | A |
| Fenamiphos sulfone | 0.02 **^1^** | 0.01 | - | Metabolite | Unclassified | NA |
| Fenamiphos sulfoxide | 0.02 **^1^** | 0.01 | - | Metabolite | Unclassified | NA |
| Fenarimol | 0.02 | 0.01 | - | Fungicide | Pyrimidine | NA |
| Fenazaquin | 0.01 | 0.01 | - | Acaricide | Quinazoline | A |
| Fenbuconazole | 0.10 | 0.01 | - | Fungicide | Triazole | A |
| Fenchlorphos | 0.01 **^2^** | 0.01 | - | Insecticide | Organophosphate | NA |
| Fenchlorphos oxon | 0.01 **^2^** | 0.01 | - | Insecticide | Organophosphate | NA |
| Fenhexamid | 0.01 | 0.01 | - | Fungicide | Fenhexamid | A |
| Fenitrothion | 0.05 | 0.01 | - | Insecticide,Acaricide | Organophosphate | NA |
| Fenoxycarb | 0.05 | 0.01 | - | Insecticide | Carbamate | A |
| Fenpiclonil | 0.01 **^3^** | 0.01 | - | Fungicide | Phenylpyrrole | NA |
| Fenpropathrin | 0.01 | 0.01 | - | Insecticide,Acaricide | Pyrethroid | NA |
| Fenpropidin | 0.10 | 0.01 | - | Fungicide | Unclassified | A |
| Fenpropimorph | 0.15 | 0.01 | - | Fungicide | Morpholine | A |
| Fenpyroximate | 0.01 | 0.01 | - | Insecticide,Acaricide | Pyrazolium | NA |
| Fenson | 0.01 **^3^** | 0.01 | - | Acaricide | Organochlorine | NA |
| Fensulfothion | 0.01 **^3^** | 0.01 | - | Insecticide,Nematicide | Organophosphate | NA |
| * LOD, limit of detection.  **^a^**, European Commission (2018) <https://ec.europa.eu/food/plant/pesticides/eu-pesticides-database/public/?event=activesubstance.selection&language=EN>; A, Approved for use in the EU; NA, not approved for use in the EU; ND, MRL not defined by the EU becasue it is not included in the EU pesticide database or is not a plant protetion product; **^b^**, PPDB (2019) <https://sitem.herts.ac.uk/aeru/ppdb/en/atoz.htm> ^1^ MRL for Fenamiphos (sum of fenamiphos and its sulphoxide and sulphone) **^2^** MRL for Fenchlorphos (sum of fenchlorphos and fenchlorphos oxon) **^3^**, Default MRL of 0.01 mg/kg according to Art 18(1)(b) Reg 396 / 2005. | | | | | | |

| ***Table S4 cont.*** List of pesticide compounds analysed in **2017** and their EU-maximum residue limit (MRL), limit of detection (LOD) in the analytical tests, product type, chemical group and approval status in the EU | | | | | | |
| --- | --- | --- | --- | --- | --- | --- |
|  | **EU MRL for** | **LOD*** | **No. of** |  |  | **Approval status** |
| **Compound** | **Wheat ^a^** | **(mg/kg)** | **positive samples** | **Type of product ^b^** | **Chemical Group ^b^** | **in the EU ^a^** |
| Fenthion | 0.01 **^1^** | 0.01 | - | Insecticide | Organophosphate | NA |
| Fenthion sulfone | 0.01 **^1^** | 0.01 | - | Insecticide | Organophosphate | NA |
| Fenthion sulfoxide | 0.01 **^1^** | 0.01 | - | Insecticide | Organophosphate | NA |
| Fenuron | 0.01 **^2^** | 0.01 | - | Herbicide | Urea | NA |
| Fenvalerate | 0.20 | 0.01 | - | Insecticide, Acaricide | Pyrethroid | NA |
| Fipronil | 0.01 | 0.01 | - | Insecticide | Phenylpyrazole | NA |
| Fiponil sulfone | 0.005 **^3^** | 0.01 | - | Insecticide | Phenylpyrazole | NA |
| Flamprop isopropyl | ND | 0.01 | - | Herbicide | Aryaminopropionic acid | ND |
| Flonicamid | 2.00 | 0.01 | - | Insecticide | Pyridinecompound | A |
| Fluazifop-P-Butyl | 0.01 **^4^** | 0.01 | - | Herbicide | Aryloxyphenoxypropionate | A |
| Fluazinam | 0.02 | 0.01 | - | Fungicide | Phenylpyridinamine | A |
| Flucythrinate | 0.01 | 0.01 | - | Insecticide | Pyrethroid | NA |
| Fludioxonil | 0.01 | 0.01 | - | Fungicide | Phenylpyrrole | A |
| Flufenacet | 0.10 | 0.01 | - | Herbicide | Oxyacetamide | A |
| Flufenoxuron | 0.05 | 0.01 | - | Insecticide | Benzoylurea | NA |
| Flumetralin | 0.01 | 0.01 | - | Plant growth regulator | Unclassified | A |
| Flumioxazin | 0.02 | 0.01 | - | Herbicide | N-phenylphtalamides | A |
| Flumorph | ND | 0.01 | - | Fungicide | Morpholine | ND |
| * LOD, limit of detection.  **^a^**, European Commission (2018) <https://ec.europa.eu/food/plant/pesticides/eu-pesticides-database/public/?event=activesubstance.selection&language=EN>; A, Approved for use in the EU; NA, not approved for use in the EU; ND, MRL not defined by the EU because it is not included in the EU pesticide database or is not a plant protection product; **^b^**, PPDB (2019) <https://sitem.herts.ac.uk/aeru/ppdb/en/atoz.htm> **^1^** MRL for Fenthion (fenthion and its oxigen analogue, their sulfoxides and sulfone expressed as parent) **^2^** Default MRL of 0.01 mg/kg according to Art 18(1)(b) Reg 396 / 2005. **^3^** MRL for Fipronil (sum of fipronil its sulfone metabolite MB46136) **^4^** MRL for Fluazifop-P (sum of all the constituent isomers of fluazifop, its esters and its conjugates) | | | | | | |

| ***Table S4 cont.*** List of pesticide compounds analysed in **2017** and their EU-maximum residue limit (MRL), limit of detection (LOD) in the analytical tests, product type, chemical group and approval status in the EU | | | | | | |
| --- | --- | --- | --- | --- | --- | --- |
|  | **EU MRL for** | **LOD*** | **No. of** |  |  | **Approval status** |
| **Compound** | **Wheat ^a^** | **(mg/kg)** | **positive samples** | **Type of product ^b^** | **Chemical Group ^b^** | **in the EU ^a^** |
| Fluometuron | 0.01 | 0.01 | - | Herbicide | Phenylurea | A |
| Fluopicolide | 0.01 | 0.01 | - | Fungicide | Benzamide | A |
| Fluopyram | 0.90 | 0.01 | - | Fungicide | Benzamide,pyramide | A |
| Fluorochloridone | ND | 0.01 | - | Herbicide | Unclassified | ND |
| Fluoxastrobin | 0.03 | 0.01 | - | Fungicide | Strobilurin | A |
| Fluquinconazole | 0.10 | 0.01 | - | Fungicide | Triazole | A |
| Fluroxypyr-1-methylheptyl ester | 0.10 | 0.01 | - | Herbicide | Pyridinecompound | A |
| Flurtamone | 0.01 | 0.01 | - | Herbicide | Pyridazinone | NA |
| Flusilazole | 0.01 | 0.01 | - | Fungicide | Triazole | NA |
| Flutolanil | 0.01 | 0.01 | - | Fungicide | Oxathiin | A |
| Flutriafol | 0.15 | 0.01 | - | Fungicide | Triazole | A |
| Fluxapyroxad | 0.40 | 0.01 | - | Fungicide | Pyrazolium | A |
| Folpet | 0.40 | 0.01 | - | Fungicide | Phthalimide | A |
| Fonophos | ND | 0.01 | - | Insecticide | Organophosphate | ND |
| Forchlorfenuron | 0.02 | 0.01 | - | Plant growth regulator | Phenylurea | A |
| Formetanate | 0.01 | 0.01 | - | Insecticide,Acaricide | Formamidine | A |
| **Formothion** | **0.01** | **0.05** | - | Insecticide,Acaricide | Organophosphate | NA |
| Fosthiasate | ND | 0.01 | - |  |  | ND |
| * LOD, limit of detection.  **^a^**, European Commission (2018) <https://ec.europa.eu/food/plant/pesticides/eu-pesticides-database/public/?event=activesubstance.selection&language=EN>; A, approved for use in the EU; NA, not approved for use in the EU; ND, MRL not defined by the EU because it is not included in the EU pesticide database or is not a plant protection product; **^b^**, PPDB (2019) <https://sitem.herts.ac.uk/aeru/ppdb/en/atoz.htm> | | | | | | |

| ***Table S4 cont.*** List of pesticide compounds analysed in **2017** and their EU-maximum residue limit (MRL), limit of detection (LOD) in the analytical tests, product type, chemical group and approval status in the EU | | | | | | |
| --- | --- | --- | --- | --- | --- | --- |
|  | **EU MRL** | **LOD*** | **No. of** |  |  | **Approval status** |
| **Compound** | **Wheat ^a^** | **(mg/kg)** | **positive samples** | **Type of product ^b^** | **Chemical Group ^b^** | **in the EU ^a^** |
| Fuberidazole | 0.05 | 0.01 | - | Fungicide | Benzimidazole | NA |
| Furalaxyl | 0.01 **^1^** | 0.01 | - | Fungicide | Acylalanine | NA |
| Furathiocarb | 0.01 | 0.01 | - | Insecticide | Carbamate | NA |
| **Glyphosate** | **10.0** | 0.01 | **21** | Herbicide | Phosphonoglycine | A |
| Haloxyfop etotyl | 0.01 | 0.01 | - | Herbicide | Aryloxyphenoxypropionate | NA |
| Haloxyfop Methyl | 0.01 | 0.01 | - | Herbicide | Aryloxyphenoxypropionate | A |
| Heptachlor | 0.01 | 0.01 | - | Insecticide | Organochlorine | NA |
| Heptachlor epoxide | 0.01 **^2^** | 0.01 | - | Metabolite | Unclassified | NA |
| Heptachlor exo Epoxide | 0.01 **^2^** | 0.01 | - |  |  | NA |
| Heptenophos | 0.01 **^1^** | 0.01 | - | Insecticide | Organophosphate | NA |
| Hexachlorobenzene | 0.01 | 0.01 | - | Fungicide | Chlorinatedhydrocarbon | NA |
| Hexachlorocyclohexane (alpha) | 0.01 | 0.01 | - | Insecticide | Organochlorine | NA |
| Hexachlorocyclohexane (beta) | 0.01 | 0.01 | - |  |  | NA |
| Hexachlorocyclohexane (delta) | ND | 0.01 | - |  |  | ND |
| Hexaconazole | 0.01 | 0.01 | - | Fungicide | Triazole | NA |
| Hexaflumuron | 0.01 **^1^** | 0.01 | - | Insecticide | Benzoylurea | NA |
| Hexazinone | 0.01 **^1^** | 0.01 | - | Herbicide | Triazinone | NA |
| Hexythiazox | 0.50 | 0.01 | - | Insecticide,Acaricide | Carboxamide | A |
| * LOD, limit of detection.  **^a^**, European Commission (2018) <https://ec.europa.eu/food/plant/pesticides/eu-pesticides-database/public/?event=activesubstance.selection&language=EN>; A, Approved for use in the EU; NA, not approved for use in the EU; ND, MRL not defined by the EU because it is not included in the EU pesticide database or is not a plant protection product; **^b^**, PPDB (2019) <https://sitem.herts.ac.uk/aeru/ppdb/en/atoz.htm> ^1^ Default MRL of 0.01 mg/kg according to Art 18(1)(b) Reg 396 / 2005. **^2^** MRL for Heptachlor (sum of heptachlor and heptachlor epoxide) | | | | | | |

| ***Table S4 cont.*** List of pesticide compounds analysed in **2017** and their EU-maximum residue limit (MRL), limit of detection (LOD) in the analytical tests, product type, chemical group and approval status in the EU | | | | | | |
| --- | --- | --- | --- | --- | --- | --- |
|  | **EU MRL for** | **LOD*** | **No. of** |  |  | **Approval status** |
| **Compound** | **Wheat ^a^** | **(mg/kg)** | **positive samples** | **Type of product ^b^** | **Chemical Group ^b^** | **in the EU ^a^** |
| Imazalil | 0.05 | 0.01 | - | Fungicide | Imidazole | A |
| Imibenconazole | 0.01 **^1^** | 0.01 | - | Fungicide | Triazole | NA |
| Imidacloprid | 0.10 | 0.01 | - | Insecticide | Neonicotinoid | A |
| Indoxacarb | 0.01 | 0.01 | - | Insecticide | Oxadiazine | A |
| Iodofenphos | 0.01 **^1^** | 0.01 | - | Insecticide | Organophosphate | NA |
| Iprodione | 0.01 | 0.01 | - | Fungicide,Nematicide | Dicarboximide | NA |
| Iprovalicarb | 0.01 | 0.01 | - | Fungicide | Carbamate | A |
| Isazofos | 0.01 **^1^** | 0.01 | - | Insecticide | Organophosphate | NA |
| Isocarbophos | 0.01 **^1^** | 0.01 | - | Acarcicide,Insecticide | Organophosphate | NA |
| Isodrin | ND | 0.01 | - | Insecticide | Cyclodiene | ND |
| Isofenphos | 0.01 **^1^** | 0.01 | - | Insecticide | Organophosphate | NA |
| Isofenphos Methyl | 0.01 **^1^** | 0.01 | - | Insecticide | Organophosphate | NA |
| isomers | ND | 0.01 | - |  |  | ND |
| Isomethiozin | ND | 0.01 | - | Herbicide | Triazinone | ND |
| Isoprocarb | 0.01 **^1^** | 0.01 | - | Insecticide | Carbamate | NA |
| Isoprothiolane | 0.01 | 0.01 | - | Fungicide | Phosphorothiolate | NA |
| Isoproturon | 0.05 | 0.01 | - | Herbicide | Urea | NA |
| Isopyrazam | 0.20 | 0.01 | - | Fungicide | Pyrazole | A |
| Isothiazolinone | ND | 0.01 | - |  |  | ND |
| * LOD, limit of detection.  **^a^**, European Commission (2018) <https://ec.europa.eu/food/plant/pesticides/eu-pesticides-database/public/?event=activesubstance.selection&language=EN>; A, Approved for use in the EU; NA, not approved for use in the EU; ND, MRL not defined by the EU because it is not included in the EU pesticide database or is not a plant protection product; **^b^**, PPDB (2019) <https://sitem.herts.ac.uk/aeru/ppdb/en/atoz.htm> **^1^** Default MRL of 0.01 mg/kg according to Art 18(1)(b) Reg 396 / 2005. | | | | | | |

| ***Table S4 cont.*** List of pesticide compounds analysed in **2017** and their EU-maximum residue limit (MRL), limit of detection (LOD) in the analytical tests, product type, chemical group and approval status in the EU | | | | | | |
| --- | --- | --- | --- | --- | --- | --- |
|  | **EU MRL for** | **LOD*** | **No. of** |  |  | **Approval status** |
| **Compound** | **wheat ^a^** | **(mg/kg)** | **positive samples** | **Type of product ^b^** | **Chemical Group ^b^** | **in the EU ^a^** |
| Isoxaben | 0.10 | 0.01 | - | Herbicide | Benzamide | A |
| Karbutilate | 0.01 **^1^** | 0.01 | - | Herbicide | Carbamate | NA |
| Kresoxim Methyl | 0.08 | 0.01 | - | Fungicide | Strobilurin | A |
| Lambda Cyhalothrin | 0.05 | 0.01 | - | Insecticide | Pyrethroid | A |
| Lenacil | 0.10 | 0.01 | - | Herbicide | Uracil | A |
| Leptophos | ND | 0.01 | - | Insecticide | Organophosphate | ND |
| Lindane | 0.01 | 0.01 | - | Insecticide,Rodenticide | Organochlorine | NA |
| Linuron | 0.01 | 0.01 | - | Herbicide | Urea | NA |
| Lufenuron | 0.01 | 0.01 | - | Insecticide | Benzoylurea | A |
| Malaoxon | 8.00 **^2^** | 0.01 | - | Insecticide,Acaricide, | Organophosphate | A |
| Malathion | 8.00 **^2^** | 0.01 |  | Insecticide,Acaricide,Veterinarysubstance | Organophosphate | A |
| Mandipropamid | 0.01 | 0.01 | - | Fungicide | Mandelamide | A |
| MCPA-thioethyl | 0.20 | 0.01 | - | Herbicide | Aryloxyalkanoicacid | A |
| Mecarbam | 0.01 | 0.01 | - | Insecticide,Acaricide | Organophosphate | NA |
| Mefenacet | 0.01 **^1^** | 0.01 | - | Herbicide | Oxyacetamide | NA |
| Mepanipyrim | 0.01 | 0.01 | - | Fungicide | Anilinopyrimidine | A |
| Mephosfolan | 0.01 **^1^** | 0.01 | - | Insecticide | Organophosphate | NA |
| Mepronil | 0.01 | 0.01 | - | Fungicide | Benzanilide | NA |
| * LOD, limit of detection.  **^a^**, European Commission (2018) <https://ec.europa.eu/food/plant/pesticides/eu-pesticides-database/public/?event=activesubstance.selection&language=EN>; A, Approved for use in the EU; NA, not approved for use in the EU; ND, MRL not defined by the EU because it is not included in the EU pesticide database or is not a plant protection product; **^b^**, PPDB (2019) <https://sitem.herts.ac.uk/aeru/ppdb/en/atoz.htm>  **^1^** Default MRL of 0.01 mg/kg according to Art 18(1)(b) Reg 396 / 2005. **^2^** MRL for Malathion (sum of malathion and malaoxon) | | | | | | |

| ***Table S4 cont.*** List of pesticide compounds analysed in **2017** and their EU-maximum residue limit (MRL), limit of detection (LOD) in the analytical tests, product type, chemical group and approval status in the EU | | | | | | |
| --- | --- | --- | --- | --- | --- | --- |
|  | **EU MRL** | **LOD*** | **No. of** |  |  | **Approval status** |
| **Compound** | **for wheat ^a^** | **(mg/kg)** | **positive samples** | **Type of product ^b^** | **Chemical Group ^b^** | **in the EU ^a^** |
| Metaflumizone | 0.05 | 0.01 | - | Insecticide | Semicarbazone | A |
| Metalaxyl | 0.01 | 0.01 | - | Fungicide | Phenylamide | A |
| Metamitron | 0.10 | 0.01 | - | Herbicide | Triazinone | A |
| Metazachlor | 0.02 | 0.01 | - | Herbicide | Chloroacetamide | A |
| Metconazole | 0.15 | 0.01 | - | Fungicide,Plant growth regulator | Triazole | A |
| Methabenzthiazuron | 0.01 | 0.01 | - | Herbicide | Urea | NA |
| Methacrifos | 0.01 | 0.01 | - | Insecticide | Organophosphate | NA |
| Methamidophos | 0.01 | 0.01 | - | Insecticide,Acaricide | Organophosphate | NA |
| Methidathion | 0.02 | 0.01 | - | Insecticide,Acaricide | Organophosphate | NA |
| Methiocarb | 0.10 **^1^** | 0.01 | - | Insecticde,Repellant | Carbamate | A |
| Methiocarb sulfone | 0.10 **^1^** | 0.01 | - | NA | Unclassified | NA |
| Methiocarb Sulfoxide | 0.10 **^1^** | 0.01 | - | NA | Carbamate | NA |
| Methomyl | 0.01 | 0.01 | - | Insecticide | Carbamate | A |
| Methoxychlor | 0.01 | 0.01 | - | Insecticide | Organochlorine | NA |
| Methoxyfenozide | 0.01 | 0.01 | - | Insecticide | Diacylhydrazine | A |
| Methyl Paraoxon | 0.02 | 0.01 | - | Insecticde,Repellant | Organophosphate | NA |
| Metobromuron | 0.01 | 0.01 | - | Herbicide | Urea | A |
| Metolachlor | 0.05 | 0.01 | - | Herbicide | Chloroacetamide | NA |
| * LOD, limit of detection.  **^a^**, European Commission (2018) <https://ec.europa.eu/food/plant/pesticides/eu-pesticides-database/public/?event=activesubstance.selection&language=EN>; A, Approved for use in the EU; NA, not approved for use in the EU; ND, MRL not defined by the EU because it is not included in the EU pesticide database or is not a plant protection product; **^b^**, PPDB (2019) <https://sitem.herts.ac.uk/aeru/ppdb/en/atoz.htm> ^1^ MRL for Methiocarb (sum of methiocarb and methiocarb sulfoxide and sulfone, expressed as methiocarb) | | | | | | |

| ***Table S4 cont.*** List of pesticide compounds analysed in **2017** and their EU-maximum residue limit (MRL), limit of detection (LOD) in the analytical tests, product type, chemical group and approval status in the EU | | | | | | |
| --- | --- | --- | --- | --- | --- | --- |
|  | **EU MRL for** | **LOD*** | **No. of** |  |  | **Approval status** |
| **Compound** | **Wheat ^a^** | **(mg/kg)** | **positive samples** | **Type of product ^b^** | **Chemical Group ^b^** | **in the EU ^a^** |
| Metolcarb | 0.01 **^1^** | 0.01 | - | Insecticide | Carbamate | NA |
| Metoxuron | 0.01 **^1^** | 0.01 | - | Herbicide | Urea | NA |
| Metrafenone | 0.07 | 0.01 | - | Fungicide | Benzophenone | A |
| Metribuzin | 0.10 | 0.01 | - | Herbicide | Triazinone | A |
| Mevinphos | 0.01 | 0.01 | - | Insecticide,Acaricide | Organophosphate | NA |
| Mirex | 0.01 **^1^** | 0.01 | - | Insecticide | Organochlorine | NA |
| Molinate | 0.01 | 0.01 | - | Herbicide | Thiocarbamate | NA |
| Monocrotophos | 0.02 | 0.01 | - | Insecticide,Acaricide | Organophosphate | NA |
| Monolinuron | 0.01 | 0.01 | - | Herbicide | Urea | NA |
| Monuron | 0.01 | 0.01 | - | Herbicide | Phenylurea | NA |
| Myclobutanil | 0.02 | 0.01 | - | Fungicide | Triazole | A |
| Napropamide | 0.05 | 0.01 | - | Herbicide | Alkanamide | A |
| Neburon | 0.01 **^1^** | 0.01 | - | Herbicide | Urea | NA |
| Nicotine | 0.01 **^1^** | 0.01 | - | Insecticide | Plantderived | NA |
| Nitenpyram | 0.01 **^1^** | 0.01 | - | Insecticide | Neonicotinoid | NA |
| Nitrofen | 0.01 | 0.01 | - | Herbicide | Diphenylether | NA |
| Nitrothal isopropyl | 0.01 **^1^** | 0.01 | - | Fungicide | Unclassified | NA |
| Novaluron | 0.01 | 0.01 | - | Insecticide | Benzoylurea | NA |
| * LOD, limit of detection.  **^a^**, European Commission (2018) <https://ec.europa.eu/food/plant/pesticides/eu-pesticides-database/public/?event=activesubstance.selection&language=EN>; A, Approved for use in the EU; NA, not approved for use in the EU; ND, MRL not defined by the EU because it is not included in the EU pesticide database or is not a plant protection product; **^b^**, PPDB (2019) <https://sitem.herts.ac.uk/aeru/ppdb/en/atoz.htm> **^1^** Default MRL of 0.01 mg/kg according to Art 18(1)(b) Reg 396 / 2005. | | | | | | |

| ***Table S4 cont.*** List of pesticide compounds analysed in **2017** and their EU-maximum residue limit (MRL), limit of detection (LOD) in the analytical tests, product type, chemical group and approval status in the EU | | | | | | |
| --- | --- | --- | --- | --- | --- | --- |
|  | **EU MRL for** | **LOD*** | **No. of** |  |  | **Approval status** |
| **Compound** | **Wheat ^a^** | **(mg/kg)** | **positive samples** | **Type of product ^b^** | **Chemical Group ^b^** | **in the EU ^a^** |
| Nuarimol | 0.01 **^1^** | 0.01 | - | Fungicide | Pyrimidine | NA |
| o,p'-DDT | 0.05 | 0.01 | - | Insecticide | Organochlorine | NA |
| Octhilinone | 0.01 **^1^** | 0.01 | - | Fungicide | Heteroaramatic | NA |
| Ofurace | 0.01 **^1^** | 0.01 | - | Fungicide | Phenylamide | NA |
| Omethoate | 0.01 | 0.01 | - | Insecticide,Acaricide | Organophosphate | NA |
| Orysastrobin | 0.01 **^1^** | 0.01 | - | Fungicide | Strobilurin | NA |
| Oxadiargyl | 0.01 | 0.01 | - | Herbicide | Oxidiazole | NA |
| Oxadiazon | 0.05 | 0.01 | - | Herbicide | Oxidiazole | NA |
| Oxadixyl | 0.01 | 0.01 | - | Fungicide | Phenylamide | NA |
| Oxamyl | 0.01 | 0.01 | - | Insecticide,Nematicide | Carbamate | A |
| Oxycarboxin | 0.01 | 0.01 | - | Fungicide | Oxathiin | NA |
| Oxydemeton-Methyl | 0.02 | 0.01 | - | Insecticide,Acaricide | Organophosphate | NA |
| Oxyfluorfen | 0.05 | 0.01 | - | Herbicide | Diphenylether | A |
| p,p-DDD | 0.05 **^2^** | 0.01 | - | Insecticide | Organochlorine | NA |
| p,p-DDE | 0.05 **^2^** | 0.01 | - | NA |  | NA |
| p,p-DDT | 0.05 **^2^** | 0.01 | - | Insecticide | Organochlorine | NA |
| Paclobutrazol | 0.01 | 0.01 | - | Plant growth regulator | Triazole | A |
| Paraoxon | ND | 0.01 | - | NA |  | ND |
| * LOD, limit of detection.  **^a^**, European Commission (2018) https://ec.europa.eu/food/plant/pesticides/eu-pesticides-database/public/?event=activesubstance.selection&language=EN; A, Approved for use in the EU; NA, not approved for use in the EU; ND, MRL not defined by the EU becasue it is not included in the EU pesticide database or is not a plant protection product; **^b^**, PPDB (2019) https://sitem.herts.ac.uk/aeru/ppdb/en/atoz.htm ^1^ Default MRL of 0.01 mg/kg according to Art 18(1)(b) Reg 396 / 2005. **^2^** MRL for DDT (sum of p,p´-DDT, o,p´-DDT, p-p´-DDE and p,p´-TDE (DDD)) | | | | | | |

| ***Table S4 cont.*** List of pesticide compounds analysed in **2017** and their EU-maximum residue limit (MRL), limit of detection (LOD) in the analytical tests, product type, chemical group and approval status in the EU | | | | | | |
| --- | --- | --- | --- | --- | --- | --- |
|  | **EU MRL for** | **LOD*** | **No. of** |  |  | **Approval status** |
| **Compound** | **Wheat ^a^** | **(mg/kg)** | **positive samples** | **Type of product ^b^** | **Chemical Group ^b^** | **in the EU ^a^** |
| Parathion ethyl | 0.02 | 0.01 | - | Insecticide,Acaricide | Organophosphate | NA |
| Parathion methyl | 0.02 | 0.01 | - | Insecticide,Repellant | Organophosphate | NA |
| Penconazole | 0.01 | 0.01 | - | Fungicide | Triazole | A |
| Pencycuron | 0.05 | 0.01 | - | Fungicide | Phenylurea | A |
| Pendimethalin | 0.05 | 0.01 | - | Herbicide | Dinitroaniline | A |
| Pentachloroaniline | ND | 0.01 | - | Metabolite | Unclassified | ND |
| Pentachlorophenol | 0.01 **^1^** | 0.01 | - | Herbicide | Organochlorine | NA |
| Pentanochlor | 0.01 **^1^** | 0.01 | - | Herbicide | Anilide | NA |
| Permethrin | 0.05 | 0.01 | - | Insecticide | Pyrethroid | NA |
| Pethoxamid | 0.01 | 0.01 | - | Herbicide | Chloroacetamide | A |
| Phenmedipham | 0.01 | 0.01 | - | Herbicide | Carbamate | A |
| Phenothrin | 0.05 | 0.01 | - | Insecticide | Pyrethroid | NA |
| Phenthoate | 0.01 **^1^** | 0.01 | - | Insecticide | Organophosphate | NA |
| Phorate | 0.02 **^2^** | 0.01 | - | Insecticide | Organophosphate | NA |
| Phorate sulfone | 0.02 **^2^** | 0.01 | - |  |  | NA |
| Phorate sulfoxide | ND | 0.01 | - | Metabolite | Organophosphate | ND |
| Phosalone | 0.01 | 0.01 | - | Insecticide, Acaricide | Organophosphate | NA |
| Phosfolan | 0.01 **^1^** | 0.01 | - | Insecticide | Organophosphate | NA |
| * LOD, limit of detection.  **^a^**, European Commission (2018) https://ec.europa.eu/food/plant/pesticides/eu-pesticides-database/public/?event=activesubstance.selection&language=EN; A, Approved for use in the EU; NA, not approved for use in the EU; ND, MRL not defined by the EU because it is not included in the EU pesticide database or is not a plant protection product; **^b^**, PPDB (2019) https://sitem.herts.ac.uk/aeru/ppdb/en/atoz.htm **^1^** Default MRL of 0.01 mg/kg according to Art 18(1)(b) Reg 396 / 2005. **^2^** MRL for Phorate (sum of phorate, its oxygen analogue and their sulfones) | | | | | | |

| ***Table S4 cont.*** List of pesticide compounds analysed in **2017** and their EU-maximum residue limit (MRL), limit of detection (LOD) in the analytical tests, product type, chemical group and approval status in the EU | | | | | | |
| --- | --- | --- | --- | --- | --- | --- |
|  | **EU MRL for** | **LOD*** | **No. of** |  |  | **Approval status** |
| **Compound** | **Wheat ^a^** | **(mg/kg)** | **positive samples** | **Type of product ^b^** | **Chemical Group ^b^** | **in the EU ^a^** |
| Phosmet | 0.05 | 0.01 | - | Insecticide | Organophosphate | A |
| Phosphamidon | 0.01 | 0.01 | - | Insecticide,Acaricide | Organophosphate | NA |
| Phoxim | 0.01 | 0.01 | - | Insecticide | Organophosphate | NA |
| Phthalimide | ND | 0.01 | - | NA |  | ND |
| Picoxystrobin | 0.01 | 0.01 | - | Fungicide | Strobilurintype-methoxyacrylate | NA |
| **Piperonyl Butoxide** | ND | 0.01 | **22** | synergist**^1^** | Cyclic aromatic | ND |
| Pirimicarb | 0.05 | 0.01 | - | Insecticide | Carbamate | A |
| Pirimicarb desmethyl | ND | 0.01 | - |  |  | ND |
| Pirimiphos Ethyl | 0.01 **^2^** | 0.01 | - | Insecticide | Organophosphate | NA |
| **Pirimiphos methyl** | **5.00** | 0.01 | **10** | Insecticide | Organophosphate | A |
| Pretilachlor | 0.01 **^2^** | 0.01 | - | Herbicide | Chloroacetamide | NA |
| Prochloraz | 0.50 | 0.01 | - | Fungicide | Imidazole | A |
| Procymidone | 0.01 | 0.01 | - | Fungicide | Dicarboximide | NA |
| Profenofos | 0.01 | 0.01 | - | Insecticide | Organophosphate | NA |
| Promecarb | 0.01 **^2^** | 0.01 | - | Insecticide | Carbamate | NA |
| Prometon | ND | 0.01 | - | Herbicide | Methoxytriazine | ND |
| Prometryn | 0.01 **^2^** | 0.01 | - | Herbicide | Triazine | NA |
| Propachlor | 0.02 | 0.01 | - | Herbicide | Chloroacetamide | NA |
| * LOD, limit of detection.  **^a^**, European Commission (2018) https://ec.europa.eu/food/plant/pesticides/eu-pesticides-database/public/?event=activesubstance.selection&language=EN; A, Approved for use in the EU; NA, not approved for use in the EU; ND, MRL not defined by the EU because it is not included in the EU pesticide database or is not a plant protection product; **^b^**, PPDB (2019) https://sitem.herts.ac.uk/aeru/ppdb/en/atoz.htm **^1^** synergist compound which has no pesticidal activity, but enhances the activity of certain pesticides such as carbamates and pyrethroids; **^2^** Default MRL of 0.01 mg/kg according to Art 18(1)(b) Reg 396 / 2005. | | | | | | |

| ***Table S4 cont.*** List of pesticide compounds analysed in **2017** and their EU-maximum residue limit (MRL), limit of detection (LOD) in the analytical tests, product type, chemical group and approval status in the EU | | | | | | |
| --- | --- | --- | --- | --- | --- | --- |
|  | **EU MRL for** | **LOD*** | **No. of** |  |  | **Approval status** |
| **Compound** | **Wheat ^a^** | **(mg/kg)** | **positive samples** | **Type of product ^b^** | **Chemical Group ^b^** | **in the EU ^a^** |
| Propamocarb | 0.01 | 0.01 | - | Fungicide | Carbamate | A |
| Propanil | 0.01 | 0.01 | - | Herbicide | Anilide | NA |
| Propaphos | 0.01 **^1^** | 0.01 | - | Insecticide | Organophosphate | NA |
| Propaquizafop | 0.05 | 0.01 | - | Herbicide | Aryloxyphenoxypropionate | A |
| Propargite | 0.01 | 0.01 | - | Acaricide | Sulphiteester | NA |
| Propazine | 0.01 **^1^** | 0.01 | - | Herbicide | Triazine | NA |
| Propetamphos | 0.01 **^1^** | 0.01 | - | Insecticide | Organophosphate | NA |
| Propham | 0.01 | 0.01 | - | Herbicide,Plant growth regulator | Carbamate | NA |
| Propiconazole | 0.09 | 0.01 | - | Fungicide | Triazole | NA |
| Propoxur | 0.05 | 0.01 | - | Insecticide | Carbamate | NA |
| Propyzamide | 0.01 | 0.01 | - | Herbicide | Benzamide | A |
| Proquinazid | 0.02 | 0.01 | - | Fungicide | Quinazolinone | A |
| Prosulfocarb | 0.01 | 0.01 | - | Herbicide | Thiocarbamate | A |
| Prothioconazole desthio | 0.10 | 0.01 | - | Fungicide | Triazolinthione | NA |
| Prothiofos | 0.01 **^1^** | 0.01 | - | Insecticide | Organophosphate | NA |
| Pymetrozine | 0.05 | 0.01 | - | Insecticide | Pyridine | NA |
| Pyraclostrobin | 0.20 | 0.01 | - | Fungicide,Plant growth regulator | Strobilurin | A |
| Pyraflufen ethyl | 0.02 | 0.01 | - | Herbicide | Phenylpyrazole | A |
| * LOD, limit of detection.  **^a^**, European Commission (2018) <https://ec.europa.eu/food/plant/pesticides/eu-pesticides-database/public/?event=activesubstance.selection&language=EN>; A, Approved for use in the EU; NA, not approved for use in the EU; ND, MRL not defined by the EU because it is not included in the EU pesticide database or is not a plant protection product; **^b^**, PPDB (2019) <https://sitem.herts.ac.uk/aeru/ppdb/en/atoz.htm>  **^1^**, Default MRL of 0.01 mg/kg according to Art 18(1)(b) Reg 396 / 2005. | | | | | | |

| ***Table S4 cont.*** List of pesticide compounds analysed in **2017** and their EU-maximum residue limit (MRL), limit of detection (LOD) in the analytical tests, product type, chemical group and approval status in the EU | | | | | | |
| --- | --- | --- | --- | --- | --- | --- |
|  | **EU MRL for** | **LOD*** | **No. of** |  |  | **Approval status** |
| **Compound** | **Wheat ^a^** | **(mg/kg)** | **positive samples** | **Type of product ^b^** | **Chemical Group ^b^** | **in the EU ^a^** |
| Pyrazophos | 0.01 | 0.01 | - | Fungicide | Phosphorothiolate | NA |
| Pyrethrin | 3.00 | 0.01 | - | Insecticide | Plantderived | A |
| Pyridaben | 0.01 | 0.01 | - | Insecticide,Acaricide | Pyridazinone | A |
| Pyridaphenthion | 0.01 **^1^** | 0.01 | - | Insecticide,Acaricide |  | NA |
| Pyrifenox | 0.01 **^1^** | 0.01 | - | Fungicide | Pyridine | NA |
| Pyrimethanil | 0.05 | 0.01 | - | Fungicide | Anilinopyrimidine | A |
| Pyrimidifen | 0.01 **^1^** | 0.01 | - |  |  | NA |
| Pyriproxyfen | 0.05 | 0.01 | - | Insecticide | Unclassified | A |
| Quinalphos | 0.01 | 0.01 | - | Insecticide | Organophosphate | NA |
| Quinoxyfen | 0.02 | 0.01 | - | Fungicide | Quinoline | NA |
| Quintozene | 0.02 | 0.01 | - | Fungicide | Chlorophenyl | NA |
| Quizalofop-ethyl | 0.05 | 0.01 | - | Herbicide | Aryloxyphenoxypropionate | NA |
| Resmethrin | 0.02 | 0.01 | - | Insecticide | Pyrethroid | NA |
| Rotenone | 0.01 | 0.01 | - | Insecticide |  | NA |
| S421 | ND | 0.01 | - |  |  | ND |
| Secbumeton | 0.01 **^1^** | 0.05 | - | Herbicide | Methoxytriazine | NA |
| Silafluofen | 0.01 **^1^** | 0.01 | - | Insecticide | Pyrethroid | NA |
| Simazine | 0.01 | 0.01 | - | Herbicide | Triazine | NA |
| * LOD, limit of detection.  **^a^**, European Commission (2018) https://ec.europa.eu/food/plant/pesticides/eu-pesticides-database/public/?event=activesubstance.selection&language=EN; A, Approved for use in the EU; NA, not approved for use in the EU; ND, MRL not defined by the EU because it is not included in the EU pesticide database or is not a plant protection product; **^b^**, PPDB (2019) https://sitem.herts.ac.uk/aeru/ppdb/en/atoz.htm **^1^**, Default MRL of 0.01 mg/kg according to Art 18(1)(b) Reg 396 / 2005. | | | | | | |

| ***Table S4 cont.*** List of pesticide compounds analysed in **2017** and their EU-maximum residue limit (MRL), limit of detection (LOD) in the analytical tests, product type, chemical group and approval status in the EU | | | | | | |
| --- | --- | --- | --- | --- | --- | --- |
|  | **EU MRL for** | **LOD*** | **No. of** |  |  | **Approval status** |
| **Compound** | **Wheat ^a^** | **(mg/kg)** | **positive samples** | **Type of product ^b^** | **Chemical Group ^b^** | **in the EU ^a^** |
| Simeconazole | 0.01 **^1^** | 0.01 | - | Fungicide | Conazole | NA |
| Spinetoram | 0.05 | 0.01 | - | Insecticide | Spinosym | A |
| Spinosad | 2.00 | 0.01 |  | Insecticide | Micro-organismderived | A |
| Spirodiclofen | 0.02 | 0.01 | - | Insecticide,Acaricide | Tetronicacid | A |
| Spiromesifen | 0.02 | 0.01 | - | Insecticide,Acaricide | Tetronicacid | A |
| Spirotetramat | 0.10 | 0.01 | - | Insecticide | Tetramicacid | A |
| Spiroxamine | 0.05 | 0.01 | - | Fungicide | Morpholine | A |
| Sulfallate | ND | 0.01 | - | Herbicide | Thiocarbamate | ND |
| Sulfentrazone | 0.01 **^1^** | 0.01 | - | Herbicide | Aryltriazolinone | NA |
| Sulprofos | 0.01 **^1^** | 0.01 | - | Insecticide | Organophosphate | NA |
| Tau-Fluvalinate | 0.05 | 0.01 | - | Insecticide | Syntheticpyrethroid | A |
| Tebuconazole | 0.30 | 0.01 | - | Fungicide | Triazole | A |
| Tebufenozide | 0.05 | 0.01 | - | Insecticide | Diacylhydrazine | A |
| Tebufenpyrad | 0.01 | 0.01 | - | Acaricide | Pyrazolium | A |
| Tebupirimiphos | ND | 0.01 | - |  |  | ND |
| Tecnazene | 0.01 | 0.01 | - | Fungicide,Plant growth regulator | Chlorophenyl | NA |
| Teflubenzuron | 0.01 | 0.01 | - | Insecticide | Benzoylurea | A |
| Tefluthrin | 0.05 | 0.01 | - | Insecticide | Pyrethroid | A |
| * LOD, limit of detection.  **^a^**, European Commission (2018) <https://ec.europa.eu/food/plant/pesticides/eu-pesticides-database/public/?event=activesubstance.selection&language=EN>; A, Approved for use in the EU; NA, not approved for use in the EU; ND, MRL not defined by the EU because it is not included in the EU pesticide database or is not a plant protection product; **^b^**, PPDB (2019) <https://sitem.herts.ac.uk/aeru/ppdb/en/atoz.htm> **^1^**, Default MRL of 0.01 mg/kg according to Art 18(1)(b) Reg 396 / 2005. | | | | | | |

| ***Table S4 cont.*** List of pesticide compounds analysed in **2017** and their EU-maximum residue limit (MRL), limit of detection (LOD) in the analytical tests, product type, chemical group and approval status in the EU | | | | | | |
| --- | --- | --- | --- | --- | --- | --- |
|  | **EU MRL for** | **LOD*** | **No. of** |  |  | **Approval status** |
| **Compound** | **Wheat ^a^** | **(mg/kg)** | **positive samples** | **Type of product ^b^** | **Chemical Group ^b^** | **in the EU ^a^** |
| Temephos | 0.01 **^1^** | 0.01 | - | Insecticide | Organophosphate | NA |
| Terbacil | 0.01 **^1^** | 0.01 | - | Herbicide | Uracil | NA |
| Terbufos | 0.01 | 0.01 | - | Insecticide | Organophosphate | NA |
| Terbufos sulfone | ND | 0.01 | - |  |  | ND |
| Terbufos sulfoxide | ND | 0.01 | - |  |  | ND |
| Terbumeton | 0.01 **^1^** | 0.01 | - | Herbicide | Triazine | NA |
| Terbuthylazine | 0.05 | 0.01 | - | Herbicide | Triazine | A |
| Terbutryn | 0.01 **^1^** | 0.01 | - | Herbicide | Triazine | NA |
| Tetrachlorvinphos | 0.01 **^1^** | 0.01 | - | Insecticide | Organophosphate | NA |
| Tetraconazole | 0.10 | 0.01 | - | Fungicide | Triazole | A |
| Tetradifon | 0.01 | 0.01 | - | Insectcide,Acaricide | Bridgeddiphenyl | NA |
| Tetraethyl dithiopyrophosphate | 0.01 **^1^** | 0.01 | - | Insecticide,Acaricide | Organophosphate | NA |
| Tetramethrin | 0.01 **^1^** | 0.01 | - | Insecticide | Pyrethroid | NA |
| Tetrasul | 0.01 **^1^** | 0.01 | - | Acaricide | Bridgeddiphenyl | NA |
| Thiabendazole | 0.01 | 0.01 | - | Fungicide | Benzimidazole | A |
| Thiacloprid | 0.10 | 0.01 | - | Insecticide | Neonicotinoid | A |
| Thiamethoxam | 0.05 | 0.01 | - | Insecticide | Neonicotinoid | A |
| Thiazafluron | 0.01 **^1^** | 0.01 | - | Herbicide | Thiadiazolylurea | NA |
| * LOD, limit of detection.  **^a^**, European Commission (2018) <https://ec.europa.eu/food/plant/pesticides/eu-pesticides-database/public/?event=activesubstance.selection&language=EN>; A, Approved for use in the EU; NA, not approved for use in the EU; ND, MRL not defined by the EU because it is not included in the EU pesticide database or is not a plant protection product; **^b^**, PPDB (2019) <https://sitem.herts.ac.uk/aeru/ppdb/en/atoz.htm> **^1^**, Default MRL of 0.01 mg/kg according to Art 18(1)(b) Reg 396 / 2005. | | | | | | |

| ***Table S4 cont.*** List of pesticide compounds analysed in **2017** and their EU-maximum residue limit (MRL), limit of detection (LOD) in the analytical tests, product type, chemical group and approval status in the EU | | | | | | |
| --- | --- | --- | --- | --- | --- | --- |
|  | **EU MRL for** | **LOD*** | **No. of** |  |  | **Approval status** |
| **Compound** | **Wheat ^a^** | **(mg/kg)** | **positive samples** | **Type of product ^b^** | **Chemical Group ^b^** | **in the EU ^a^** |
| Thidiazuron | 0.01 **^1^** | 0.01 | - | Plant growth regulator | Phenylurea | NA |
| Thiobencarb | 0.01 | 0.01 | - | Herbicide | Thiocarbamate | NA |
| Thiocyclam | 0.01 **^1^** | 0.01 | - | Insectcide | Unclassified | NA |
| Thiodicarb | 0.01 | 0.01 | - | Insectcide | Carbamate | NA |
| Thiofanox | 0.01 **^1^** | 0.01 | - | Insectcide | Carbamate | NA |
| Thiometon | 0.01 **^1^** | 0.01 | - | Insectcide,Acaricide | Organophosphate | NA |
| Thiophanate Methyl | 0.05 | 0.01 | - | Fungicide | Benzimidazole | A |
| Tolclofos methyl | 0.01 | 0.01 | - | Fungicide | Chlorophenyl | A |
| Tolylfluanid | 0.05 | 0.01 | - | Fungicide,Acaricide | Sulphamide | NA |
| Triadimefon | 0.01 | 0.01 | - | Fungicide | Triazole | NA |
| Triadimenol | 0.10 | 0.01 | - | Fungicide | Triazole | A |
| Triallate | ND | 0.01 | - | Herbicide | Thiocarbamate | ND |
| Triazamate | 0.01 **^1^** | 0.01 | - | Insectcide | Carbamoyltriazole | NA |
| Triazophos | 0.02 | 0.01 | - | Insectcide,Acaricide | Organophosphate | NA |
| Tribenuron methyl | 0.01 | 0.01 | - | Herbicide | Sulfonylurea | NA |
| Trichlorfon | 0.01 | 0.01 | - | Insectcide | Organophosphate | NA |
| Tridemorph | 0.01 | 0.01 | - | Fungicide | Morpholine | NA |
| Trietazine | 0.01 **^1^** | 0.01 | - | Herbicide | Triazine | NA |
| * LOD, limit of detection.  **^a^**, European Commission (2018) <https://ec.europa.eu/food/plant/pesticides/eu-pesticides-database/public/?event=activesubstance.selection&language=EN>; A, Approved for use in the EU; NA, not approved for use in the EU; ND, MRL not defined by the EU because it is not included in the EU pesticide database or is not a plant protection product; **^b^**, PPDB (2019) <https://sitem.herts.ac.uk/aeru/ppdb/en/atoz.htm> **^1^**, Default MRL of 0.01 mg/kg according to Art 18(1)(b) Reg 396 / 2005. | | | | | | |

| ***Table S4 cont.*** List of pesticide compounds analysed in **2017** and their EU-maximum residue limit (MRL), limit of detection (LOD) in the analytical tests, product type, chemical group and approval status in the EU | | | | | | |
| --- | --- | --- | --- | --- | --- | --- |
|  | **EU MRL for** | **LOD*** | **No. of** |  |  | **Approval status** |
| **Compound** | **Wheat ^a^** | **(mg/kg)** | **positive samples** | **Type of product ^b^** | **Chemical Group ^b^** | **in the EU ^a^** |
| Trifloxystrobin | 0.30 | 0.01 | - | Fungicide | Strobilurin | A |
| Trifloxysulfuron | 0.01 **^1^** | 0.01 | - | Herbicide | Sulfonylurea | NA |
| Triflumizole | 0.02 | 0.01 | - | Fungicide | Imidazole | A |
| Triflumuron | 0.01 | 0.01 | - | Insectcide | Benzoylurea | A |
| Trifluralin | 0.01 | 0.01 | - | Herbicide | Dinitroaniline | NA |
| Triflusulfuron-methyl | 0.01 | 0.01 | - | Herbicide | Sulfonylurea | A |
| Triforine | 0.01 | 0.01 | - | Fungicide,Acaricide | Piperazine | NA |
| Triticonazole | 0.01 | 0.01 | - | Fungicide | Triazole | A |
| Uniconazole | 0.01 **^1^** | 0.01 | - | Plant growth regulator | Triazole | NA |
| Vamidothion | 0.01 **^1^** | 0.01 | - | Insectcide,Acaricide | Organophosphate | NA |
| Vernolate | 0.01 **^1^** | 0.01 | - | Herbicide | Thiocarbamate | NA |
| Vinclozolin | 0.01 | 0.01 | - | Fungicide | Oxazole | NA |
| Zoxamide | 0.02 | 0.01 | - | Fungicide | Benzamide | A |
| * LOD, limit of detection.  **^a^**, European Commission (2018) <https://ec.europa.eu/food/plant/pesticides/eu-pesticides-database/public/?event=activesubstance.selection&language=EN>; A, Approved for use in the EU; NA, not approved for use in the EU; ND, MRL not defined by the EU because it is not included in the EU pesticide database or is not a plant protection product; **^b^**, PPDB (2019) <https://sitem.herts.ac.uk/aeru/ppdb/en/atoz.htm> **^1^**, Default MRL of 0.01 mg/kg according to Art 18(1)(b) Reg 396 / 2005. | | | | | | |

| ***Table S5.*** Interactions means ± SE for the effects of species and farming systems on percentage (%) of positive samples detected with piperonyl butoxide. | | | |
| --- | --- | --- | --- |
|  | **Factor 1** | **Factor 2** | |
|  |  | **Farming System** | |
| **Crop protection products (CPPs)** | **Species** | **Conventional** | **Organic** |
| **piperonyl butoxide** | Spelt Wheat | 41±17 Aa | 22±9 Aa |
|  | Common Wheat | 30 ± 7 Aa | 3 ±2 Bb |
| For each parameter assessed means labelled with capital letter within the same row or the same lower-case letter within the same column are not significant different (Tukey’s honestly significant difference test P<0.05); | | | |

| ***Table S6.***  Main effect means ± SE and **nonparametric analysis** (Mann-Whitney U Test) result for the effects of year (2016 and 2017), country (Germany and UK), wheat species (common vs Spelt wheat), farming system (organic vs conventional) and flour type (white vs wholemeal) on the on concentrations of crop protection product (CCP) residues. | | | | | | |
| --- | --- | --- | --- | --- | --- | --- |
|  | **CPP residue concentration (µg/kg)** | | | | | |
| **Factor** | **deltamethrin** | **chlormequat** | **piperonyl butoxide** | **glyphosate** | **pirimiphos methyl** | **2-phenylphenol** |
| Year |  |  |  |  |  |  |
| 2016 (n=143) | 5.8 ±0.4 | 17.5 ±2.5 | 17.5 ±5.6 | 25.0±0.0 | 5.1 ±0.1 | 5.0 ±0.0 |
| 2017 (n=117) | 6.1 ±0.6 | 38.4 ±4.5 | 19.8 ±6.3 | 48.5±6.5 | 6.1 ±0.6 | 8.1 ±0.9 |
| **Country** |  |  |  |  |  |  |
| Germany (n= 121) | 5.4 ±0.2 | 24.0 ±4.4 | 10.4 ±2.3 | 27.6±1.9 | 5.5 ±0.3 | 8.8 ±1.1 |
| UK (n=139) | 6.5 ±0.7 | 33.9 ±3.6 | 26.2 ±7.8 | 47.5±6.7 | 5.8 ±0.5 | 5.0 ±0.0 |
| **Species** |  |  |  |  |  |  |
| Spelt wheat (n=85) | 5.0 ±0.0 | 16.5 ±3.0 | 7.9 ±1.1 | 28.8±2.6 | 5.0 ±0.0 | 5.0 ±0.0 |
| Common wheat (n=175) | 6.5 ±0.6 | 35.5 ±3.9 | 24.2 ±6.4 | 42.9±5.4 | 6.0 ±0.5 | 7.6 ±0.8 |
| **Farming system** |  |  |  |  |  |  |
| Conventional (n=125) | 7.1 ±0.8 | 55.0 ±5.0 | 33.2 ±8.8 | 52.6±7.6 | 6.3 ±0.7 | 8.7 ±1.1 |
| Organic (n=135) | 5.0 ±0.0 | 5.5 ±0.3 | 5.5 ±0.3 | 25.0±0.0 | 5.1 ±0.1 | 5.0 ±0.0 |
| **Flour type** |  |  |  |  |  |  |
| White (n=151) | 5.5 ±0.3 | 21.9 ±2.3 | 12.9 ±2.7 | 32.4±2.8 | 5.5 ±0.2 | 8.0 ±0.9 |
| Wholemeal (n=109) | 6.7 ±0.9 | 39.6 ±5.8 | 27.1 ±9.6 | 46.5±8.0 | 6.0 ±0.7 | 5.0 ±0.0 |
| **Nonparametric Analysis** |  |  |  |  |  |  |
| **(p-value)** |  |  |  |  |  |  |
| Year | NS | **0.0002** | NS | **<0.0001** | NS | **0.0019** |
| Country | NS | **0.0003** | **0.0438** | **0.0004** | NS | **0.0001** |
| Species | **0.0251** | **0.0018** | NS | **0.0190** | **0.0102** | **0.0137** |
| Farming System | **0.0008** | **<0.0001** | **<0.0001** | **<0.0001** | **0.0011** | **0.0002** |
| Flour Type | NS | NS | NS | NS | NS | **0.0026** |

| 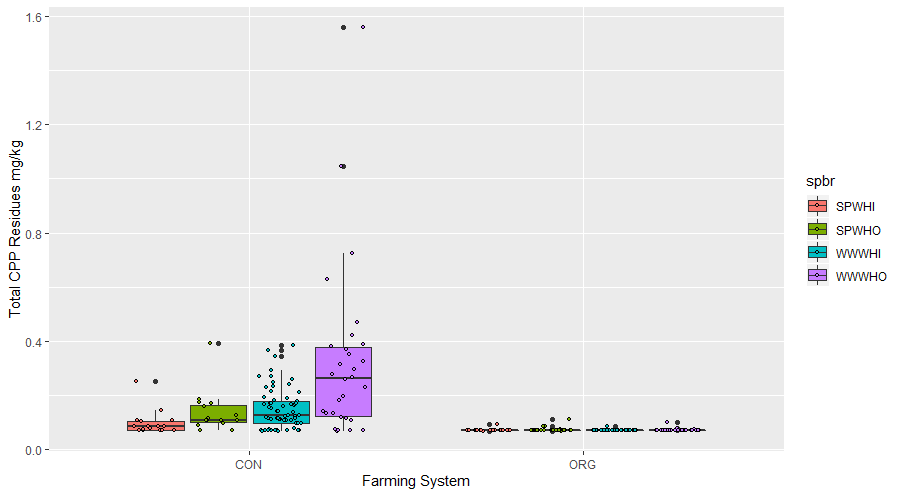 |
| --- |
| ***Figure S1.*** Boxplots for **total crop protection product (CPP)** residue concentrations (mg/kg) in white and wholegrain, conventional and organic, common and Spelt wheat flour (data from all samples collected in the UK and Germany in 2016 and 2017). CON: conventional, ORG: organic; spbr: species and flour types; SPWHI: white spelt wheat flour, SPWHO: wholegrain spelt wheat flour; WWWHI: white common wheat flour, WWWHO: wholegrain common wheat flour; concentrations in individual samples are shown as small circles (**⭘**). The total CPP residue included residues detected for deltamethrin, chlormequat, piperonyl butoxide, pirmiphos methyl, 2-phenyl-phenol, glyphosate, chlorpyrifos methyl, cypermethrin, pendimethalin, tebuconazole. Residues below the limit of detection (LOD) were estimated as half the LOD. |

| 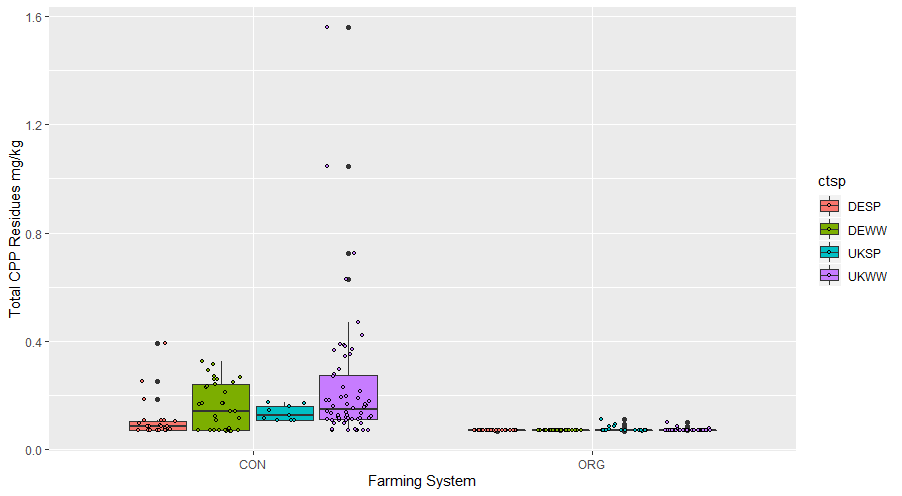 |
| --- |
| ***Figure S2.*** Boxplots for **total crop protection product (CPP)** residue concentrations (mg/kg) in conventional and organic common and spelt wheat flour from the UK and Germany (data from all white and wholegrain samples collected in 2016 and 2017). CON: conventional; ORG: organic; ctsp: country and species; DESP: German spelt wheat flour; DEWW: German common wheat flour; UKSP: UK spelt wheat flour; UKWW: UK common wheat flour; concentrations in individual samples are shown as *small circles (****⭘****)*. The total CPP residue included residues detected for deltamethrin, chlormequat, piperonyl butoxide, pirmiphos methyl, 2-phenyl-phenol, glyphosate, chlorpyrifos methyl, cypermethrin, pendimethalin, tebuconazole. Residues below the limit of detection (LOD) were estimated as half the LOD. |

| 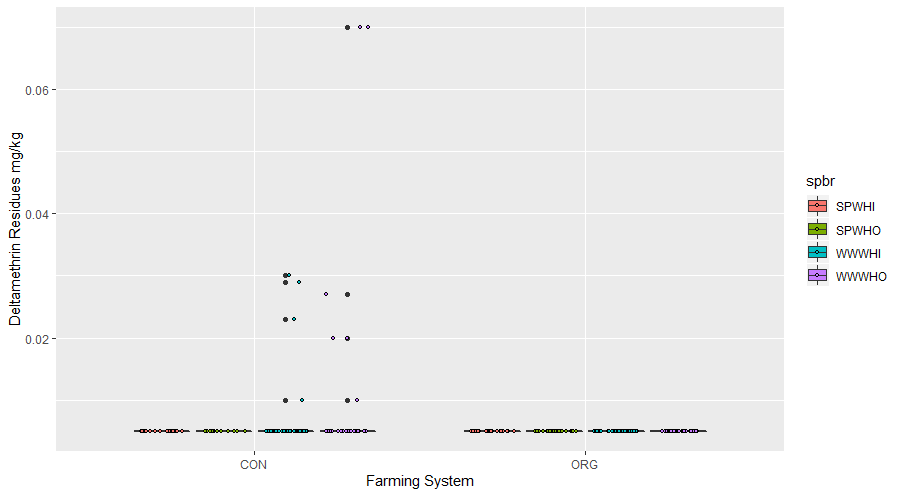 |
| --- |
| **Figure S3.** Boxplots for **deltamethrin** residue concentrations (mg/kg) in white and wholegrain, conventional and organic, common and Spelt wheat flour (data from all samples collected in the UK and Germany in 2016 and 2017). CON: conventional, ORG: organic; spbr: species and flour types; SPWHI: white spelt wheat flour, SPWHO: wholegrain spelt wheat flour; WWWHI: white common wheat flour, WWWHO: wholegrain common wheat flour; concentrations in individual samples are shown as small circles (**⭘**). |

| 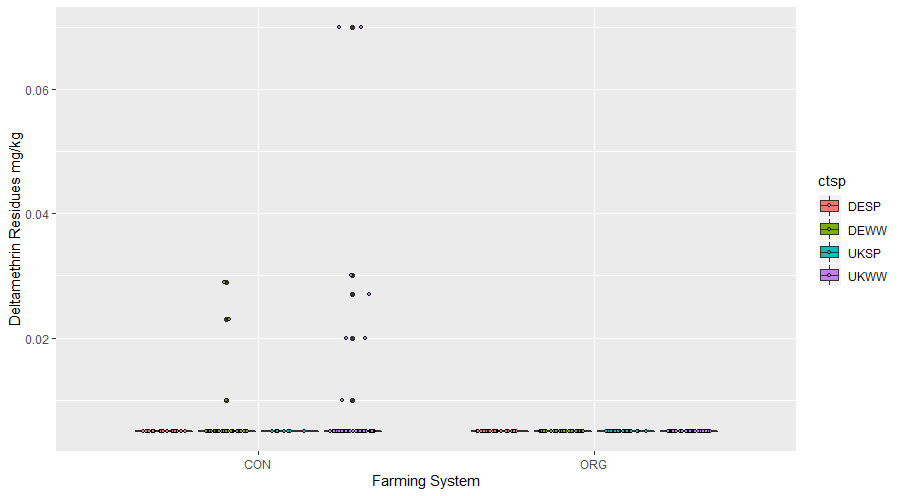 |
| --- |
| **Figure S4.** Boxplots for **deltamethrin** residue concentrations (mg/kg) in conventional and organic common and spelt wheat flour from the UK and Germany (data from all white and wholegrain samples collected in 2016 and 2017). CON: conventional; ORG: organic; ctsp: country and species; DESP: German spelt wheat flour; DEWW: German common wheat flour; UKSP: UK spelt wheat flour; UKWW: UK common wheat flour; concentrations in individual samples are shown as small circles (**⭘**). |

| 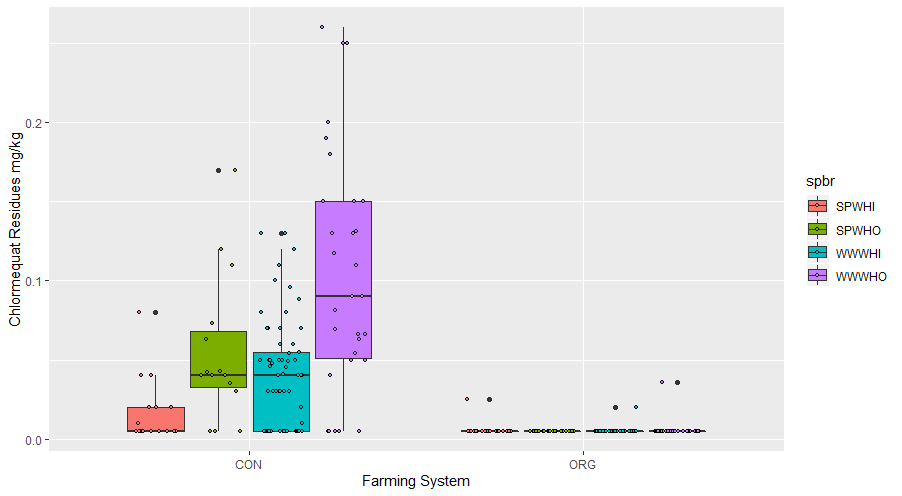 |
| --- |
| **Figure S5.** Boxplots for **chlormequat** residue concentrations (mg/kg) in white and wholegrain, conventional and organic, common and Spelt wheat flour (data from all samples collected in the UK and Germany in 2016 and 2017). CON: conventional, ORG: organic; spbr: species and flour types; SPWHI: white spelt wheat flour, SPWHO: wholegrain spelt wheat flour; WWWHI: white common wheat flour, WWWHO: wholegrain common wheat flour; concentrations in individual samples are shown as small circles (⭘). |

| 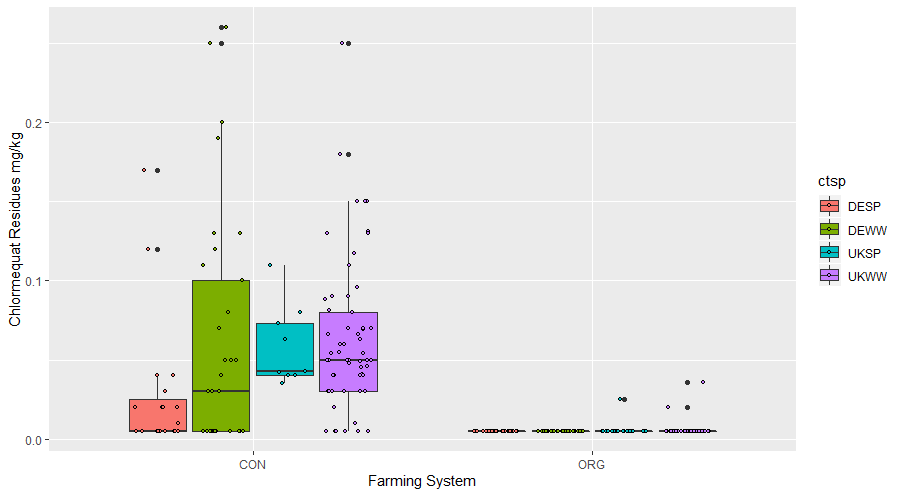 |
| --- |
| **Figure S6.** Boxplots for **chlormequat** residue concentrations (mg/kg) in conventional and organic common and spelt wheat flour from the UK and Germany (data from all white and wholegrain samples collected in 2016 and 2017). CON: conventional; ORG: organic; ctsp: country and species; DESP: German spelt wheat flour; DEWW: German common wheat flour; UKSP: UK spelt wheat flour; UKWW: UK common wheat flour; concentrations in individual samples are shown as small circles (**⭘**). |

| 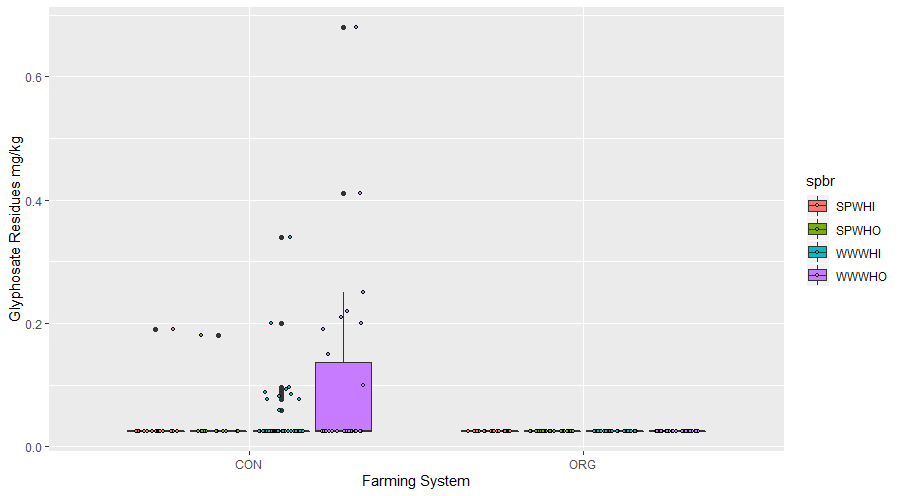 |
| --- |
| **Figure S7.** Boxplots for **glyphosate** residue concentrations (mg/kg) in white and wholegrain, conventional and organic, common and Spelt wheat flour (data from all samples collected in the UK and Germany in 2016 and 2017). CON: conventional, ORG: organic; spbr: species and flour types; SPWHI: white spelt wheat flour, SPWHO: wholegrain spelt wheat flour; WWWHI: white common wheat flour, WWWHO: wholegrain common wheat flour; concentrations in individual samples are shown as small circles (**⭘**). |

| 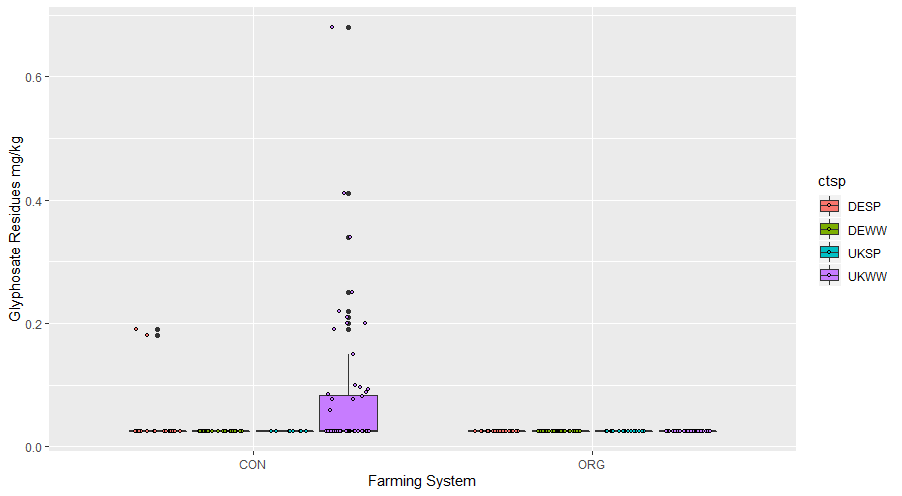 |
| --- |
| **Figure S8.** Boxplots for **glyphosate** residue concentrations (mg/kg) in conventional and organic common and spelt wheat flour from the UK and Germany (data from all white and wholegrain samples collected in 2016 and 2017). CON: conventional; ORG: organic; ctsp: country and species; DESP: German spelt wheat flour; DEWW: German common wheat flour; UKSP: UK spelt wheat flour; UKWW: UK common wheat flour; concentrations in individual samples are shown as small circles (**⭘**). |

| 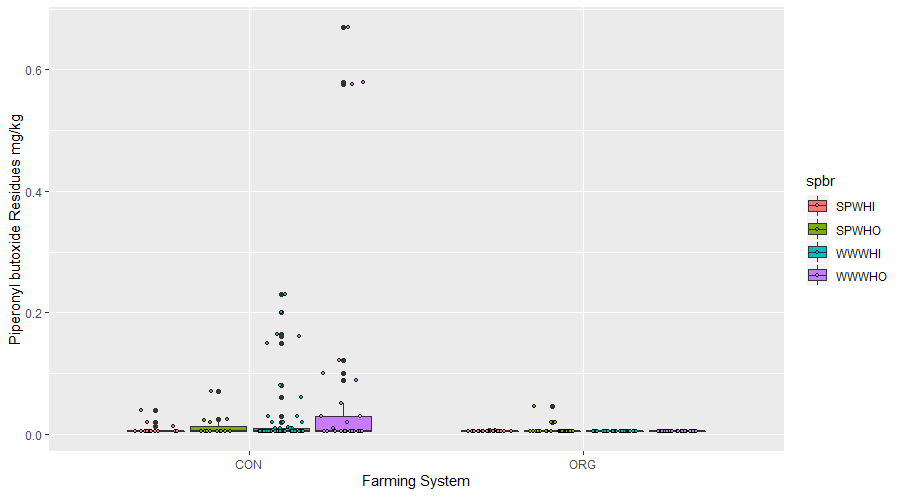 |
| --- |
| **Figure S9.** Boxplots for **piperonyl butoxide** residue concentrations (mg/kg) in white and wholegrain, conventional and organic, common and Spelt wheat flour (data from all samples collected in the UK and Germany in 2016 and 2017). CON: conventional, ORG: organic; spbr: species and flour types; SPWHI: white spelt wheat flour, SPWHO: wholegrain spelt wheat flour; WWWHI: white common wheat flour, WWWHO: wholegrain common wheat flour; concentrations in individual samples are shown as small circles (**⭘**). |

| 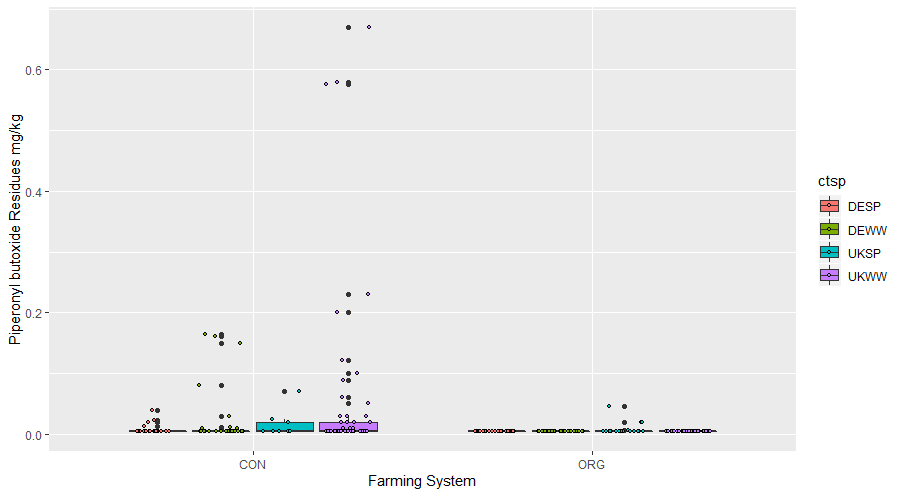 |
| --- |
| **Figure S10.** Boxplots for **piperonyl butoxide** residue concentrations (mg/kg) in conventional and organic common and spelt wheat flour from the UK and Germany (data from all white and wholegrain samples collected in 2016 and 2017). CON: conventional; ORG: organic; ctsp: country and species; DESP: German spelt wheat flour; DEWW: German common wheat flour; UKSP: UK spelt wheat flour; UKWW: UK common wheat flour; concentrations in individual samples are shown as small circles (**⭘**). |

| 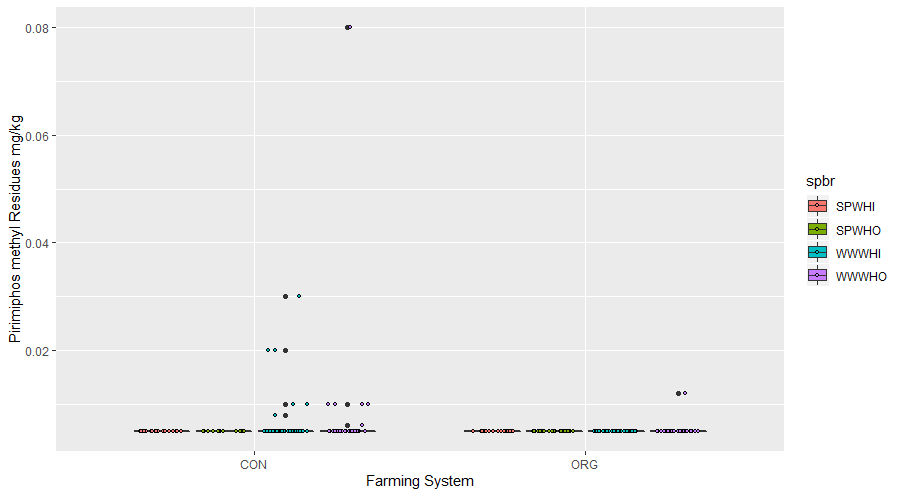 |
| --- |
| **Figure S11.** Boxplots for **pirimiphos methyl** residue concentrations (mg/kg) in white and wholegrain, conventional and organic, common and Spelt wheat flour (data from all samples collected in the UK and Germany in 2016 and 2017). CON: conventional, ORG: organic; spbr: species and flour types; SPWHI: white spelt wheat flour, SPWHO: wholegrain spelt wheat flour; WWWHI: white common wheat flour, WWWHO: wholegrain common wheat flour; concentrations in individual samples are shown as small circles (**⭘**). |

| 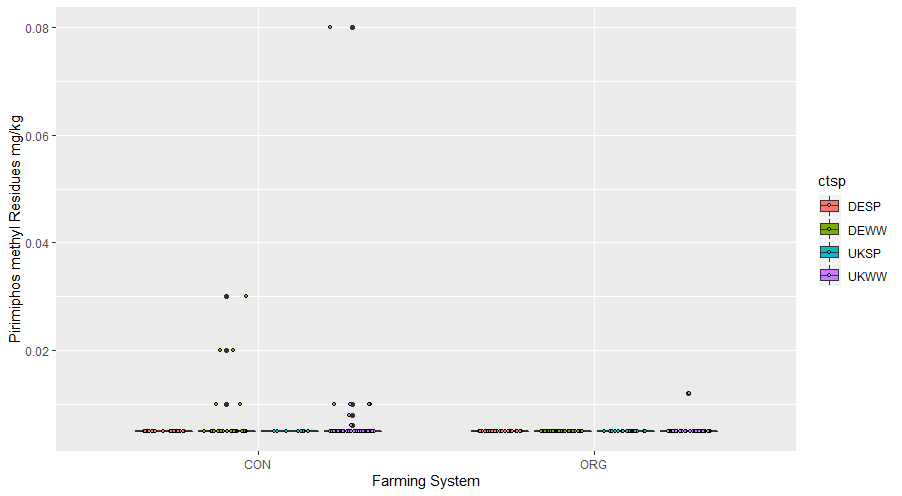 |
| --- |
| **Figure S12.** Boxplots for **pirimiphos methyl** residue concentrations (mg/kg) in conventional and organic common and spelt wheat flour from the UK and Germany (data from all white and wholegrain samples collected in 2016 and 2017). CON: conventional; ORG: organic; ctsp: country and species; DESP: German spelt wheat flour; DEWW: German common wheat flour; UKSP: UK spelt wheat flour; UKWW: UK common wheat flour; concentrations in individual samples are shown as small circles (**⭘**). |

| 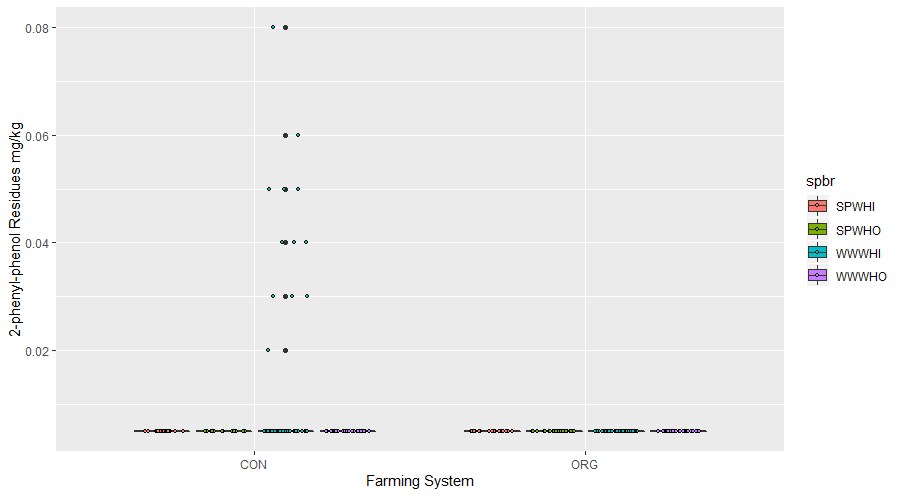 |
| --- |
| **Figure S13.** Boxplots for **2-phenyl-phenol** residue concentrations (mg/kg) in white and wholegrain, conventional and organic, common and Spelt wheat flour (data from all samples collected in the UK and Germany in 2016 and 2017). CON: conventional, ORG: organic; spbr: species and flour types; SPWHI: white spelt wheat flour, SPWHO: wholegrain spelt wheat flour; WWWHI: white common wheat flour, WWWHO: wholegrain common wheat flour; concentrations in individual samples are shown as small circles (**⭘**). |

| 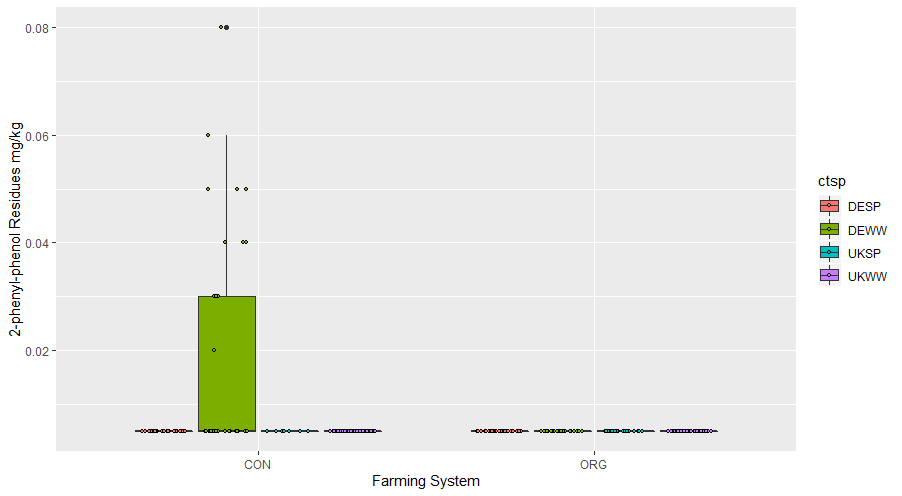 |
| --- |
| **Figure S14.** Boxplots for **2-phenyl-phenol** residue concentrations (mg/kg) in conventional and organic common and spelt wheat flour from the UK and Germany (data from all white and wholegrain samples collected in 2016 and 2017). CON: conventional; ORG: organic; ctsp: country and species; DESP: German spelt wheat flour; DEWW: German common wheat flour; UKSP: UK spelt wheat flour; UKWW: UK common wheat flour; concentrations in individual samples are shown as small circles (**⭘**). |

**References**

Anagnostopoulos, C. J., Aplada Sarli, P., Miliadis, G. E., & Haroutounian, C. A. (2010). Validation of the QuEChERS method for the determination of 25 priority pesticide residues in cereal-based baby foods by gas chromatography with electron capture and nitrogen phosphorous detection. *Hellenic Plant Protection Journal, 3*(2), 71-80.

Anagnostopoulos, C. J., Liapis, K., Haroutounian, S., & Paspatis, E. (2013). Simultaneous determination of different classes of plant growth regulator in high water content agricultural products by liquid chromatography tandem mass spectrometry and time of flight mass spectrometry. *Journal of Liquid Chromatography & Related Technologies, 36*(3), 315-335.

Anagnostopoulos, C. J., & Miliadis, G. E. (2009). Method validation for the determination of pesticide residues in wheat flour by gas chromatography. *Hellenic Plant Protection*, 15.

Anastassiades, M., Kolberg, D., Benkenstein, A., Zechmann, S., Mack, D., Barth, A., . . . Dörk, D. (Producer). (2016). Quick Method for the Analysis of Residues of numerous Highly Polar Pesticides in Food Commodities involving Simultaneous Extraction with Methanol and Determination via LC-MS/MS (QuPPe-AO-Method) (Version 2). Retrieved from <http://www.cromlab.es/Articulos/Metodos/EU/meth_QuPPe_AO%20(V2%20010116).pdf>

Danezis, G. P., Anagnostopoulos, C. J., Liapis, K., & Koupparis, M. A. (2016). Multi-residue analysis of pesticides, plant hormones, veterinary drugs and mycotoxins using HILIC chromatography–MS/MS in various food matrices. *Analytica Chimica Acta, 942*, 121-138.

European Commission (Producer). (2007). CRL for Single Residue Methods: Analysis of Acidic Pesticides in Wheat Flour Samples by LC-MS(/MS) using the QuEChERS method. Retrieved from <http://www.eurl-pesticides.eu/library/docs/cf/acidicpesticides_wheat_quechers.pdf>
